# Regulation of HLTF-mediated PCNA polyubiquitination by RFC and PCNA monoubiquitination levels determines choice of damage tolerance pathway

**DOI:** 10.1093/nar/gky943

**Published:** 2018-10-18

**Authors:** Yuji Masuda, Satoshi Mitsuyuki, Rie Kanao, Asami Hishiki, Hiroshi Hashimoto, Chikahide Masutani

**Affiliations:** 1 Department of Genome Dynamics, Research Institute of Environmental Medicine, Nagoya University, Furo-cho, Chikusa-ku, Nagoya 464-8601, Japan; 2 Nagoya University Graduate School of Medicine, 65 Tsurumai-cho, Showa-ku, Nagoya 466-8550, Japan; 3 School of Pharmaceutical Sciences, University of Shizuoka, 52-1 Yada, Suruga-ku, Shizuoka 422-8002, Japan

## Abstract

DNA-damage tolerance protects cells via at least two sub-pathways regulated by proliferating cell nuclear antigen (PCNA) ubiquitination in eukaryotes: translesion DNA synthesis (TLS) and template switching (TS), which are stimulated by mono- and polyubiquitination, respectively. However, how cells choose between the two pathways remains unclear. The regulation of ubiquitin ligases catalyzing polyubiquitination, such as helicase-like transcription factor (HLTF), could play a role in the choice of pathway. Here, we demonstrate that the ligase activity of HLTF is stimulated by double-stranded DNA via HIRAN domain-dependent recruitment to stalled primer ends. Replication factor C (RFC) and PCNA located at primer ends, however, suppress *en bloc* polyubiquitination in the complex, redirecting toward sequential chain elongation. When PCNA in the complex is monoubiquitinated by RAD6-RAD18, the resulting ubiquitin moiety is immediately polyubiquitinated by coexisting HLTF, indicating a coupling reaction between mono- and polyubiquitination. By contrast, when PCNA was monoubiquitinated in the absence of HLTF, it was not polyubiquitinated by subsequently recruited HLTF unless all three-subunits of PCNA were monoubiquitinated, indicating that the uncoupling reaction specifically occurs on three-subunit-monoubiquitinated PCNA. We discuss the physiological relevance of the different modes of the polyubiquitination to the choice of cells between TLS and TS under different conditions.

## INTRODUCTION

DNA-damage tolerance (DDT) pathways protect cells from a wide variety of endogenous and exogenous genotoxic agents by recovering stalled DNA replication caused by insult to DNA. At least two sub-pathways regulated by proliferating cell nuclear antigen (PCNA) ubiquitination at the conserved lysine residue K164 exist in humans (1,2), translesion DNA synthesis (TLS) and template switching (TS). TLS is stimulated by PCNA monoubiquitination catalyzed by an E2-E3 complex, RAD6-RAD18 (3–5), and is potentially error-prone because of the miscoding nature of most damaged nucleotides, whereas TS is theoretically accurate (error-free). TS is promoted by K63-linked polyubiquitination of PCNA catalyzed by the combined actions of the RAD6-RAD18 complex and another E3–E2 pair, such as helicase-like transcription factor (HLTF) and MMS2-UBC13 (1,6,7).

HLTF is a human homologue of the SWI/SNF-related ubiquitin ligase RAD5 of the yeast *Saccharomyces cerevisiae* (6,7). HLTF/RAD5 is a multi-functional protein consisting of multiple domains. The HIRAN (HIP116, Rad5p N-terminal) domain (8) is located at the N-terminal, and the RING domain is inside the large SWI/SNF helicase domain. HIRAN is a 3′-OH-binding-module, and its biochemical activity is required for replication fork reversal together with the SWI/SNF helicase domain (9–15). The RING domain is required for the polyubiquitination of PCNA (6,7,16–18), and is involved in the monoubiquitination of PCNA (19). In addition, HLTF catalyzes D-loop formation without requiring ATP binding and/or hydrolysis (20). As a transcription factor, HLTF controls many genes involved in a variety of cellular processes through its capacity to specifically bind to DNA sequences (21).

TLS and TS operate differently at each cell stage depending on the type of DNA lesion and the level of damage. Yeast genetics has provided extensive evidence and insights. In response to chronic low-dose ultraviolet (CLUV) irradiation (0.18 J m^−2^ min^−1^), TS is the predominant pathway, and the contribution of TLS is negligible for survival. Defects in TS are not rescued by the remaining TLS (22), indicating that TLS and TS are not interchangeable. The possibility that TS precedes TLS was proposed based on experiments in which cells exposed to acute methyl methanesulfonate (MMS) treatment (0.033%, 30 min) were released into S phase (23). However, another study with CLUV showed a synergistic effect in TLS- and TS-deficient mutants, indicating that TLS and TS are interchangeable for survival (24). Under exposure to low-dose MMS (0.001%), cells have a preference for TS, which operates earlier, whereas TLS is executed later. Under such conditions, defects in TS are rescued by TLS and *vice versa*, demonstrating that the pathways are interchangeable (25). However, in cells exposed to acute UV irradiation with 10 J m^−2^ in G1 phase, TLS operates predominantly in the subsequent S phase, and TS functions marginally for survival. Defects in TLS are not rescued by the remaining TS (26). Although the relative contribution of TLS and TS differs under the respective experimental conditions, these studies demonstrate that DDT is not required for bulk genome replication, because DDT-deficient cells can replicate through damaged DNA without delay despite their arrest at G2 (22–26). Limiting TLS or TS to the G2 phase promotes damage tolerance, indicating that DDT acts on gaps left behind the replication fork (postreplicative) (23,25,26). The postreplicative nature of DDT is also demonstrated by visualization of gap-repair synthesis and sister chromatid junctions (26–28). In contrast to the above observations, TS-deficient cells that are released from G1 in the presence of 0.033% MMS progress slowly through S phase, whereas such delay is not observed in TLS-deficient cells. This suggests that TS functions during DNA replication in the DDT to relatively high-dose MMS-induced DNA damage (29). Impediment to DNA replication in TS-deficient cells is also observed in cells exposed to acute adozelesin treatment (30). This impediment is also observed in DDT-deficient cells in response to acute UV irradiation with 40 J m^−2^ in G1 phase (26). In mammalian cells, TLS operates potentially in both the S and G2 phases. The S-phase mode plays a role in fork progression, and the G2-phase mode is required for gap-filling after bulk genome replication (31–35). The UV hyper-mutability of pol η-deficient human cells implies that TLS is predominant in the response to cyclobutane pyrimidine dimer (CPD), and the lesions are not channeled to TS (36,37). By contrast, overexpression of K63R mutant ubiquitin and downregulation of pol η synergistically sensitize human cells to UV irradiation, suggesting that a DDT pathway stimulated by K63-linked ubiquitination and a TLS pathway involving pol η are interchangeable for survival (38). TS promoted by PCNA polyubiquitination has not been clearly demonstrated in mammalian cells; however, the presence of a homology-dependent repair (HDR) pathway has been demonstrated by which DNA fragments containing defined lesions are integrated into the chromosome (39,40). In mammalians, TLS or HDR is selected depending on the type of DNA lesion and the clustering of closely opposed lesions, supporting that TLS and HDR are interchangeable (39,40). These results from yeast to humans indicate that, in certain situations, the selection of TLS or TS is defined at the time of onset and the pathways are not interchangeable, whereas under specific conditions, TLS and TS are interchangeable. Despite these extensive analyses, the molecular basis determining the choice between TLS and TS remains to be determined.

We previously provided biochemical evidence that HLTF is a DNA-dependent ubiquitin ligase that generates a ubiquitin chain on UBC13 of the complex with MMS2 (18) by seesaw reactions (41). HLTF does not transfer the ubiquitin chain to monoubiquitinated PCNA, but rather to the ubiquitin moiety of RAD6∼Ub of the complex with RAD18 (18). Polyubiquitination of PCNA is mediated by direct transfer of the resulting thiol-linked ubiquitin chain on RAD6 to unmodified PCNA in a reaction catalyzed by RAD18 (18). This result suggested the existence of a mechanism via which TLS and TS are independently regulated (18). By contrast, other studies showed that monoubiquitinated PCNA is the substrate of RAD5 for subsequent polyubiquitination (16,17), indicating that TLS can be channeled to TS. The biochemical regulation of the two different modes of PCNA polyubiquitination, *en bloc* chain transfer and sequential chain elongation, remains to be clarified.

In the present study, we elucidated the regulatory mechanism underlying the ligase activity of HLTF. The results demonstrated that the polyubiquitination of PCNA by HLTF is mediated by three different pathways determined by replication factor C (RFC) and the levels of PCNA monoubiquitination. Based on the biochemical properties of HLTF identified in the study, we discuss the physiological relevance of the different modes of polyubiquitination for the choice between TLS and TS in different cellular situations.

## MATERIALS AND METHODS

### Proteins

E1, MMS2-UBC13, RAD6-(RAD18)_2_, RAD6-(^his^RAD18)_2_, HLTF, ^his^HLTF, ubiquitin, replication protein A (RPA), PCNA, RFC and their mutants were purified as described previously (18,42–46). Three-subunit-monoubiquitinated PCNA and partially monoubiquitinated PCNA with histidine-tagged ubiquitin were prepared as described previously (18,47). Protein concentrations were determined using the Bio-Rad protein assay with BSA (Bio-Rad) as the standard.

A truncated mutant of the HIRAN domain consisting of 230–1009 amino acid residues was purified as a histidine-tagged protein (^his^HLTF^ΔN^). Column chromatography was performed at 4°C on an AKTA system (GE Healthcare Life Science) using columns from GE Healthcare. The truncated mutant was cloned into pBAD22A (obtained from the National BioResource Project, National Institute of Genetics, Mishima, Japan) with the N-terminal histidine-tagged sequence of pET15 (Novagen), resulting in pBAD-hisHLTFΔN. BL21 (DE3) (48) harboring pBAD-hisHLTFΔN was grown in 2 l of SB medium (49) supplemented with ampicillin (250 μg/ml) at 15°C. The protein was induced with 1% l-(+)-arabinose for 20 h, and then purified by sequential chromatography on Ni^2+^-charged HiTrap chelating HP, HiTrap Heparin HP, and Superdex 200 columns. The peak fraction containing ^his^HLTF^ΔN^ was frozen in liquid nitrogen and stored at −80°C.

Hybrid PCNA consisting of ^his^PCNA^K164R^ and PCNA was purified as follows: fragments encoding PCNA and the histidine-tagged PCNA(K164R) mutant were cloned into pET21 (Novagen) and pACYCDuet-1 (Novagen), yielding pET21-PCNA and pACYC-hisPCNA(K164R), respectively. The N-terminally histidine-tagged sequence was obtained from pET15. BL21 (DE3) harboring pET21-PCNA and pACYC-hisPCNA(K164R) was grown in 4 l of LB supplemented with ampicillin (250 μg/ml) and chloramphenicol (30 μg/ml) at 15°C. The proteins were induced with 0.2 mM IPTG for 8 h, and then purified by sequential chromatography on Ni^2+^-charged HiTrap chelating HP, HiTrap Q FF and Superdex 200 columns. The peak fraction, which contained both ^his^PCNA^K164R^ and PCNA, was frozen in liquid nitrogen and stored at −80°C.

GST-fusion proteins were purified as follows: GST-fusion genes were cloned into pET21 (Novagen). BL21 (DE3) harboring each plasmid was grown in 2 l of LB supplemented with ampicillin (250 μg/ml) at 15°C. The protein was induced with 0.2 mM IPTG for 7 h and then purified by sequential chromatography on GSTrap FF and Superdex 200 columns. The peak fraction was frozen in liquid nitrogen and stored at −80°C.

### DNA

Poly(dA) and poly(dT) were purchased from The Midland Certified Reagent Company Inc. (No. 10782 and No. 10774, respectively). To generate poly(dA)-oligo(dT), poly(dA) and synthetic 18-mer oligo(dT) were mixed at a 2:1 ratio of nucleotide amounts. M13mp18 single-stranded (ss)DNA, M13mp18 RF I, and lambda DNA were purchased from Takara Bio Inc. Multiple-primed M13mp18 ssDNA was generated by annealing 20 synthetic 36-mer oligonucleotides (Supplementary Table S1), and an amount corresponding to 150 pmol nucleotides of M13mp18 ssDNA backbone (0.02 pmol) containing 0.41 pmol of the 3′-OH was used in each assay unless otherwise indicated.

### Western blotting

Reaction products were analyzed by western blotting. Anti-ubiquitin monoclonal antibody (Santa Cruz Biotechnology, Ub [P4D1] sc-8017), anti-PCNA monoclonal antibody (Santa Cruz Biotechnology, PCNA [PC10] sc-56), anti-RFC1 polyclonal antibody (Santa Cruz Biotechnology, RFC1 [H300] sc-20993), anti-HLTF monoclonal antibody (Abcam, [EPR14761] ab183042), and anti-HLTF serum raised in a rabbit against a histidine-tagged N-terminal fragment of HLTF (1–148 amino acids) were used. In co-immunoprecipitation assays, proteins were detected using anti-RFC1 polyclonal antibody (Abcam, ab3556), anti-HLTF polyclonal antibody (Abcam, ab17984), and anti-FLAG M2 monoclonal antibody (SIGMA-ALDRICH, F1804). Proteins were separated on SDS 4–20% gradient or 15% polyacrylamide gels, blotted onto a PVDF membrane, and probed with the indicated antibodies. Signals were detected with a Chemi-Lumi One L kit (Nacalai Tesque, 07880-70) using the ImageQuant™ LAS 4000 Mini Biomolecular Imager (GE Healthcare) and analyzed using ImageJ 1.48v software (NIH).

### A quantification system of ubiquitin molecules in chains

To make a standard, a mixture of K63-linked ubiquitin chains was generated by HLTF. A reaction mixture (300 μl) containing 20 mM HEPES–NaOH (pH 7.5), 50 mM NaCl, 0.02 mg/ml BSA, 1 mM dithiothreitol (DTT), 10 mM MgCl_2_, 1 mM ATP, E1 (10 pmol), MMS2-UBC13 (190 pmol), ubiquitin (2 nmol) and HLTF (63 pmol) was preincubated at 30°C for 2 min, and the reaction was initiated by addition of poly(dA)-oligo(dT) (1.8 nmol as nucleotides) for 10 min. The reactions were terminated by addition of 300 μl of sample-loading buffer [100 mM Tris–HCl (pH 6.8), 200 mM DTT, 4% sodium dodecyl sulfate (SDS), 0.1% bromophenol blue, 20% glycerol] for SDS-PAGE and heating at 95°C for 5 min. The amount of total ubiquitin in the mixture of chains was determined using a K63-linked tetra-ubiquitin chain (Boston Biochem, UC310) as the standard. Both the mixture of K63-linked ubiquitin chains and the tetra-ubiquitin chain were used as standards. To quantify ubiquitin molecules in chains in reaction samples, various amounts of the standard chains were loaded next to the samples and analyzed by western blotting.

### Ubiquitin chain-formation assay by HLTF

The reaction mixture (25 μl) contained 20 mM HEPES–NaOH (pH 7.5), 50 mM NaCl, 0.02 mg/ml BSA, 1 mM DTT, 10 mM MgCl_2_, 1 mM ATP, E1 (0.85 pmol), MMS2-UBC13 (16 pmol), ubiquitin (174 pmol), HLTF (1.3 pmol), and DNA unless otherwise indicated. Reaction mixtures were prepared on ice and then incubated at 30°C for 10 min unless otherwise indicated. The reactions were terminated by addition of 25 μl of sample-loading buffer [100 mM Tris–HCl (pH 6.8), 200 mM DTT, 4% SDS, 0.1% bromophenol blue, and 20% glycerol] for SDS-PAGE. For non-reducing conditions, reactions were terminated by addition of urea-SDS buffer [50 mM Tris–HCl (pH 6.8), 4% SDS, 4 M urea, 0.1% bromophenol blue, and 10% glycerol] followed by incubation at 37°C for 30 min before loading gels. Products were analyzed by western blotting.

### HLTF binding to M13mp18 ss or double-stranded (ds) DNA tethered to magnetic beads

The 5′-biotinylated primer (CTCTCTCTCTCTCTCTCTCTCAGGGTTTTCCCAGTCACGACGTTGTAAAACGACGG) was annealed to ss M13mp18 DNA. To convert ss M13mp18 DNA into dsDNA, 650 ng of ss M13mp18 DNA annealed to the biotinylated primer was incubated with 10 U of T7 DNA polymerase (New England Biolabs) in a reaction buffer containing 0.33 mM each of dGTP, dATP, dTTP and dCTP at 37°C for 15 min. The reaction was terminated by addition of EDTA, and the enzyme was inactivated at 75°C for 20 min. DNA was recovered by precipitation with isopropanol. An amount corresponding to 150 pmol nucleotides as ss M13mp18 DNA or 300 pmol nucleotides as ds M13mp18 DNA was immobilized onto a 10 μl suspension of streptavidin magnetic beads M280 (Dynabeads) in DNA-binding buffer [20 mM HEPES–NaOH (pH 7.5), 50 mM NaCl, 0.2 mg/ml BSA, 1 mM DTT, and ubiquitin (174 pmol)] by incubation at room temperature for 30 min, followed by two washes with the same buffer of 25 μl each using a Dynal magnet. Note that ubiquitin prevented non-specific protein binding to the beads. After incubation with HLTF (1.3 pmol) in buffer (25 μl) containing 20 mM HEPES–NaOH (pH 7.5), 50 mM NaCl, 0.2 mg/ml BSA, 1 mM DTT, 10 mM MgCl_2_, 1 mM ATP, and ubiquitin (174 pmol) at 4°C for 2 min, bound and unbound fractions were separated using the Dynal magnet and analyzed by western blotting with an anti-HLTF antibody.

### Assembly of proteins on multiply primed M13mp18 ssDNA tethered to magnetic beads

The 5′-biotinylated primer was annealed to the multiply primed ss M13mp18 DNA. An amount corresponding to 150 pmol nucleotides of ss M13mp18 DNA backbone containing 0.43 pmol of 3′-OH was immobilized onto a 10 μl suspension of streptavidin magnetic beads M280 in DNA-binding buffer by incubation at room temperature for 30 min and chilled on ice. The buffer condition was then adjusted in the reaction mixture (25 μl) to 20 mM HEPES–NaOH (pH 7.5), 50 mM NaCl, 0.2 mg/ml BSA, 1 mM DTT, 10 mM MgCl_2_, 1 mM ATP, RPA (7.3 pmol), PCNA (1.0 pmol trimer), RFC (1.2 pmol), and ubiquitin (174 pmol) unless otherwise indicated. After incubation at 30°C for 1.5 min, reactions were terminated with 1.2 μl of 500 mM EDTA, and the mixtures were immediately chilled on ice. Subsequent washes to remove unbound proteins were performed at 4°C using the Dynal magnet with 40 μl of wash buffer [20 mM HEPES–NaOH (pH 7.5), 0.6 M NaCl, 0.2 mg/ml BSA, and 1 mM DTT] four times. Then, the beads were incubated in a reaction mixture (25 μl) containing 20 mM HEPES–NaOH (pH 7.5), 50 mM NaCl, 0.2 mg/ml BSA, 1 mM DTT, 10 mM MgCl_2_, 1 mM ATP, HLTF (1.3 pmol) and ubiquitin (174 pmol) at 4°C for 2 min, and washed with wash buffer four times. The beads were then incubated in a reaction mixture (25 μl) containing 20 mM HEPES–NaOH (pH 7.5), 50 mM NaCl, 0.2 mg/ml BSA, 1 mM DTT, 10 mM MgCl_2_, 1 mM ATP, E1 (0.85 pmol), MMS2-UBC13 (16 pmol), RAD6-(RAD18)_2_ (0.62 pmol), and ubiquitin (174 pmol) at 30°C for 5 min unless otherwise indicated. The reactions were terminated by addition of sample-loading buffer (25 μl), and products were analyzed by western blotting.

### Polyubiquitination of PCNA in bulk reaction

The reaction mixture (25 μl) contained 20 mM HEPES–NaOH (pH 7.5), 50 mM NaCl, 0.2 mg/ml BSA, 1 mM DTT, 10 mM MgCl_2_, 1 mM ATP, E1 (0.85 pmol), RAD6-(RAD18)_2_ (0.62 pmol), MMS2-UBC13 (16 pmol), ubiquitin (174 pmol), HLTF (1.3 pmol) and poly(dA)-oligo(dT) (150 pmol as nucleotides) unless otherwise indicated. Reaction mixtures were prepared on ice, and then incubated at 30°C for 10 min unless otherwise indicated. The reactions were terminated by addition of sample-loading buffer (25 μl). Products were analyzed by western blotting.

### DNA replication assay

DNA polymerase assays were performed as described previously (46). The standard reaction mixture (25 μl) contained 20 mM HEPES–NaOH (pH 7.5); 50 mM NaCl; 0.2 mg/ml BSA; 1 mM DTT; 10 mM MgCl_2_; 1 mM ATP; 0.1 mM each of dGTP, dATP, dTTP, and [α-^32^P]dCTP; 33 fmol (240 pmol for nucleotides) of singly primed ss M13mp18 DNA with the 36-mer primer CAGGGTTTTCCCAGTCACGACGTTGTAAAACGACGG; RPA (9.1 pmol); PCNA (1.0 pmol trimer); RFC (0.26 pmol); polymerase δ (pol δ; 0.38 pmol); E1 (0.85 pmol); MMS2-UBC13 (16 pmol); RAD6-(RAD18)_2_ (0.62 pmol); ubiquitin (174 pmol); and the indicated amounts of HLTF. Reaction mixtures lacking pol δ were preincubated at 30°C for 1 min, and reactions were started by addition of pol δ. After incubation at 30°C for 10 min, reactions were terminated with 2 μl of 300 mM EDTA, and the mixtures were immediately chilled on ice. Samples (5 μl) were spotted on DE81 paper (Whatman), which was washed three times with 0.5 M Na_2_HPO_4_. The amount of incorporated [α-^32^P]dCMP into DNA was determined as the radioactivity retained on the paper. For electrophoretic analysis of replication products, 5 μl samples were mixed with 1 μl of loading buffer (150 mM NaOH/10 mM EDTA/6% sucrose/0.1% bromophenol blue) and electrophoresed on 0.7% alkaline–agarose gels as described previously (50).

### Co-immunoprecipitation

A *HLTF*-disrupted U-2 OS cell (*HLTF*^−/−^) was generated by the CRISPR/Cas9 system. Mutations in both alleles (one and two base deletions) as well as no HLTF signal by western blotting were confirmed (data not shown). Then, stable cell lines expressing 3× FLAG-tagged *HLTF* (*HLTF*^−/−^/*^3xFLAG^HLTF*) and a vector-transfected cell line (*HLTF*^−/−^/vector) were established. The cell lines were maintained in Dulbecco's modified Eagle's medium (Wako) supplemented with 10% fetal bovine serum, 1× penicillin-streptomycin solution (Nacalai Tesque), and 0.4 mg/ml G418 at 37°C with 5% CO_2_. After one wash with phosphate-buffered saline, cells were irradiated with UVC (15 J/m^2^), incubated under the above-described conditions for 3 h, and harvested. The cells were suspended in lysis buffer [20 mM Tris–HCl (pH 7.6), 150 mM KCl, 10% glycerol, 0.5% NP-40, 1.5 mM MgCl_2_, 0.2 μg/ml antipain, 0.2 μg/ml aprotinin, 0.1 μg/ml leupeptin, 0.08 μg/ml pepstatin, 0.05 mM EGTA and 0.25 mM phenylmethylsulfonyl fluoride (PMSF)], and soluble materials were separated by centrifugation. The precipitates (insoluble fraction) were suspended in micrococcal nuclease buffer [20 mM Tris–HCl (pH 7.6), 100 mM KCl, 0.3 M sucrose, 0.1% Triton X-100, 2 mM MgCl_2_, 1 mM CaCl_2_ and cOmplete™ EDTA-free Protease Inhibitor Cocktail (Roche)] and digested with MNase at 25°C for 10 min. The reaction was terminated by addition of EDTA, and the MNase-soluble fraction (chromatin fraction) was collected by centrifugation. The chromatin fraction was incubated with anti-FLAG affinity gel (Sigma) at 4°C for 2 h, washed three times with wash buffer [20 mM Tris–HCl (pH 7.6), 150 mM KCl, 10% glycerol, 0.2% Triton X-100, 5 mM MgCl_2_, 0.2 mM EDTA, 0.2 mM β-mercaptoethanol and 0.2 mM PMSF] and eluted with 0.2 mg/ml 3× FLAG peptide in wash buffer.

### Pull-down assays

For a pull-down assay of PCNA with histidine-tagged proteins, a 3 μl suspension of Profinity™ IMAC Ni-Charged Resin (BIO-RAD, #1560131) was resuspended in 10 μl of binding buffer containing 20 mM HEPES–NaOH (pH 7.5), 50 mM NaCl, 10 mM imidazole, 0.2 mg/ml BSA and 1 mM DTT, and then incubated on ice for 5 min with 10 pmol of each protein. After washing the beads twice with 50 μl of the binding buffer, 2.5 pmol of PCNA was introduced and incubated on ice for 5 min in 25 μl of binding buffer. After washing the beads three times with 50 μl of binding buffer, proteins bound to the beads were analyzed by western blotting with anti-PCNA antibody. After detection of the signals, the membrane was stained with Coomassie Brilliant Blue (CBB) to visualize the immobilized histidine-tagged proteins.

For a pull-down assay of RFC with histidine-tagged proteins, a 4 μl suspension of Profinity™ IMAC Ni-Charged Resin was resuspended in 10 μl of binding buffer containing 20 mM HEPES–NaOH (pH 7.5), 300 mM NaCl, 10 mM imidazole, 0.2 mg/ml BSA, 0.1% Triton X-100 and 1 mM DTT, and then incubated on ice for 5 min with 10 pmol of each protein. After washing the beads twice with 50 μl of binding buffer, 2.6 pmol of RFC was introduced and incubated on ice for 10 min in 25 μl of binding buffer. After washing the beads three times with 50 μl of binding buffer, proteins bound to the beads were analyzed by western blotting with anti-RFC1 antibody. After detection of the signals, the membrane was stained with CBB to visualize the immobilized histidine-tagged proteins.

For GST-pull-down assays, an 80 μl suspension of Glutathione Sepharose™ 4 Fast Flow (GE Healthcare, 17-5132-01) was washed with 500 μl of equilibration buffer containing 20 mM HEPES–NaOH (pH 7.5), 200 mM NaCl, 0.2 mg/ml BSA and 1 mM DTT, and then incubated at 4°C for 30 min with 27 pmol of each GST-fusion protein in 100 μl of equilibration buffer. After washing the beads four times with 400 μl of binding buffer containing 20 mM HEPES–NaOH (pH 7.5), 50 mM NaCl, and 1 mM DTT, 10 pmol of PCNA or ^his^HLTF was incubated at 4°C for 5 min in 100 μl of binding buffer. After washing the beads twice with 300 μl of binding buffer, the beads were incubated with 20 μl of elution buffer containing 20 mM HEPES–NaOH (pH 7.5), 300 mM NaCl, 1 mM DTT, and 10 mM glutathione at 4°C for 30 min. The eluted proteins were analyzed by western blotting. After detection of the signals, the membrane was stained with CBB to visualize the immobilized GST-fusion proteins.

### ATPase assay

The reaction mixture (25 μl) contained 20 mM HEPES–NaOH (pH 7.5), 50 mM NaCl, 0.2 mg/ml BSA, 1 mM DTT, 10 mM MgCl_2_, 1 mM [γ-^32^P]ATP, poly(dA)-oligo(dT) (150 pmol as nucleotides), and the indicated amounts of HLTF. Reactions were initiated by addition of the enzyme, and incubated at 30°C for 90 min. The reaction products (2 μl each) were immediately spotted onto a thin-layer chromatography plate of polyethyleneimine cellulose (Merck), and developed with 1 M formic acid and 0.3 M LiCl. The amount of remaining ATP was calculated from the ratio of [γ-^32^P]ATP to total [γ-^32^P]ATP plus inorganic phosphate [^32^P], and the amount of hydrolysis was determined by subtraction of the remaining ATP from the initial amount of ATP at time 0.

## RESULTS

### Quantification of ubiquitin chain synthesis catalyzed by HLTF

**Figure 1.**
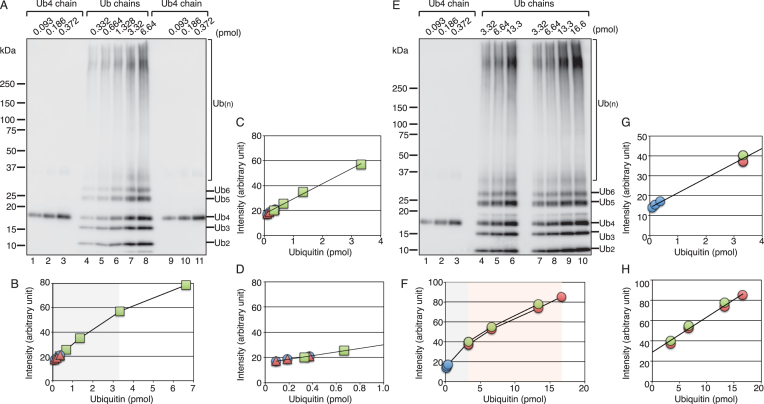
A quantification system of ubiquitin molecules in chains. (**A, E**) Western blot analysis of standard ubiquitin chains. The indicated amounts of ubiquitin molecules in chains were loaded. (**B–D**) The relative signal intensity in each lane of (**A**) was plotted in a graph. Data from lanes 1–3, 4–8 and 9–11 are indicated as circles, squares, and triangles, respectively. (**F–H**) The relative signal intensity in each lane of (**E**) was plotted in a graph. Data from lanes 1–3, 4–6 and 7–10 are represented as blue, green, and red circles, respectively. The ranges used for quantification, one for low amounts (0.093–3.32 pmol of ubiquitin) and one for high amounts (3.32–16.6 pmol of ubiquitin), are shown as shadowing and coloring in the background, respectively, in (B) and (F). ‘Ub_4_ chain’ and ‘Ub chains’ indicate the standards, K63-linked tetra-ubiquitin chain and a mixture of K63-linked ubiquitin chains, respectively.

We previously demonstrated that the ligase activity of HLTF is strongly stimulated by DNA (18), indicating that DNA is a regulatory factor for HLTF. To analyze the DNA-dependent activation mechanism, we established a quantification system of ubiquitin in the chains. First, a mixture of Lys63-linked ubiquitin chains was generated through a HLTF-mediated reaction (MATERIALS AND METHODS and see also the next paragraph for details of the reaction). Second, the total amount of ubiquitin in the chains generated by HLTF from dimer to gel top was determined by western blotting with an anti-ubiquitin antibody using commercially available Lys63-linked tetra-ubiquitin as the standard (data not shown). Third, various amounts of tetra-ubiquitin (Figure 1A, lanes 1–3 and 9–11) and the mixture of chains generated (Figure 1A, lanes 4–8) were subjected to western blotting to determine the dynamic range as the standard. The signal intensities from dimer to gel top of each lane were plotted in a graph (Figure 1B–D), which showed a linear correlation between the signal intensities and loading amounts between 0.093 pmol of ubiquitin in the tetra-ubiquitin chain and 3.32 pmol of ubiquitin in the mixture of chains (Figure 1B, range of shadowing in the background, and Figure 1C and D). Figure 1E shows another result obtained using larger amounts of the mixture of chains. The quantification results confirmed the linear correlation from 0.093 pmol of ubiquitin in the tetra-ubiquitin chain to 3.32 pmol in the mixture of chains (Figure 1F, range of shadowing in the background, and Figure 1G). Another linear correlation was observed between 3.32 and 16.6 pmol of ubiquitin in the mixture of chains (Figure 1F, range of coloring in the background, and Figure 1H). Based on these results, the amounts of ubiquitin in chains were measured using two standard curves, one for low amounts (0.093–3.32 pmol of ubiquitin) and one for high amounts (3.32–16.6 pmol of ubiquitin) of ubiquitin in chains.

**Figure 2.**
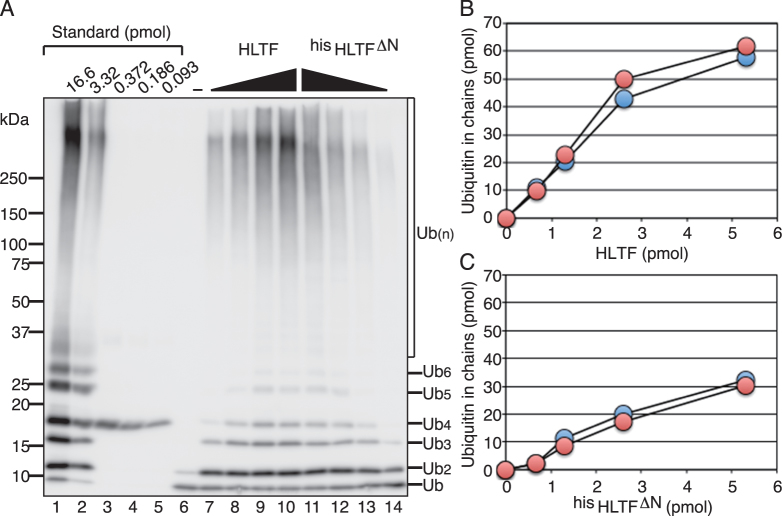
Titrations of HLTF and ^his^HLTF^ΔN^. (**A**) Representative western blot data for quantification. Chain-formation activities were analyzed under standard assay conditions containing E1, MMS2-UBC13, and ubiquitin at 30°C for 10 min with 150 pmol of poly(dA)-oligo(dT) nucleotides and increasing amounts of enzymes in the order of 0.7, 1.3, 2.6 and 5.3 pmol. One-tenth of the amount of the product was loaded with the indicated amount of standards. (B, C) The total amounts of ubiquitin in chains in each 25 μl reaction mixture with the indicated amounts of HLTF (**B**) and ^his^HLTF^ΔN^ (**C**) were plotted. Data obtained from two independent western blots are shown.

The method was first applied to titration assays of HLTF (Figure 2A). In the presence of E1, MMS2-UBC13, ubiquitin, and DNA, HLTF forms thiol-linked ubiquitin chains on UBC13 and then transfers the chains to free ubiquitin in solution, generating a mixture of ubiquitin chains linked to UBC13 and unanchored ubiquitin chains in solution (18). Hereafter, the ubiquitin ligase activity generating UBC13-linked and unanchored ubiquitin chains is described as chain-formation activity. Treatment of the reaction products with standard SDS-PAGE sample-loading buffer containing a reducing agent to break thiol-linkages results in a mixture of only unanchored ubiquitin chains (18). In the following assays, the total amounts of ubiquitin in the unanchored ubiquitin chains generated by treatment with the reducing agent were quantified to measure the chain-formation activity. After a 10 min reaction with poly(dA)–oligo(dT) as a source of DNA, 1/10 of the total reaction products were loaded on the gel together with the standards (Figure 2A), and the amounts of ubiquitin in the chains from dimer to gel top were measured. The total amounts of ubiquitin in chains in a 25 μl reaction mixture were plotted (Figure 2B). Quantification data were obtained from two independent western blots (Figure 2B). The results showed that the catalytic velocity of the reaction was 1.67 ± 0.14 min^−1^ under the reaction conditions used.

Because HIRAN is one of the conserved motifs in HLTF/RAD5 family proteins (8), it could be one of the regulatory factors for the ligase activity of HLTF. To determine whether HIRAN was involved in the chain-formation activity, a truncated mutant of the HIRAN domain was purified as a histidine-tagged protein (^his^HLTF^ΔN^) (Figure 5D and Supplementary Figure S1A), and its activity was analyzed. The results of the titration experiment are shown in Figure 2A and C. Quantification data were obtained from two independent western blots (Figure 2C). The catalytic velocity of ^his^HLTF^ΔN^ was 0.69 ± 0.10 min^−1^, which was 2–3-fold lower than that of wild-type HLTF. These results confirmed that the quantification method was reproducible; quantification data were hereafter obtained from at least two independent western blots for each assay, and the average was plotted. A previous report concluded that deletion of HIRAN does not affect the ligase activity of HLTF (11); however, the assay system used to analyze the production of polyubiquitinated PCNA in the presence of RAD6-RAD18 with a double-stranded pUC19 plasmid nicked by the BstNBI enzyme is not suitable to detect the difference. In the following sections, we provide evidence of the role of HIRAN in the ligase reactions catalyzed by HLTF.

### DNA containing the 3′-OH primer end most efficiently stimulates the chain-formation activity of HLTF

**Figure 3.**
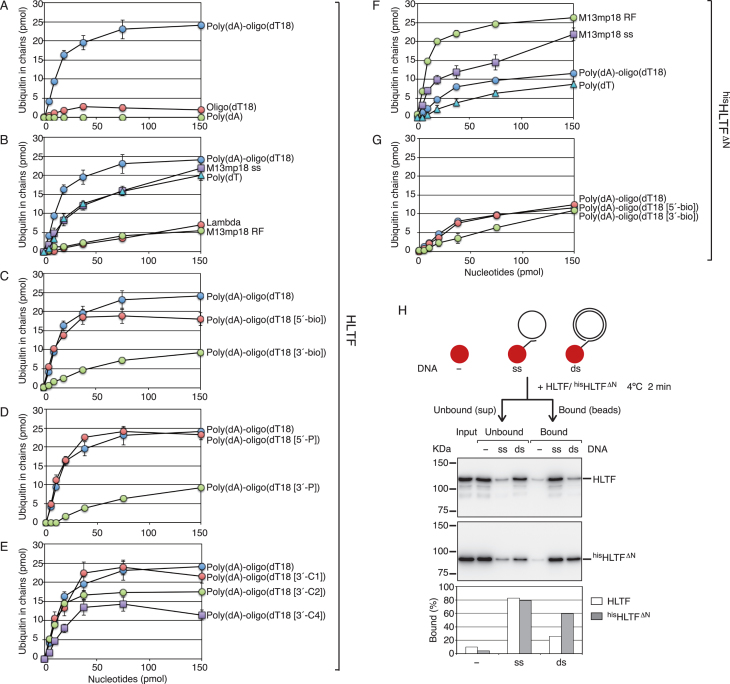
Effects of various types of DNA. Chain-formation activities of wild-type HLTF (**A**–**E**) and ^his^HLTF^ΔN^ (**F, G**) were analyzed under standard assay conditions containing E1, MMS2-UBC13, and ubiquitin at 30°C for 10 min with the indicated DNA. The total amounts of ubiquitin in chains in each 25 μl reaction mixture were plotted. (A) Poly(dA)-oligo(dT) is a 2:1 mixture of poly(dA) and 18-mer oligo(dT) as nucleotides. (B, F) The indicated DNA was titrated as shown. (C, G) Poly(dA) was annealed to 18-mer oligo(dT) modified with biotin at the 5′- or 3′-end at a 2:1 ratio as nucleotides. (D) Poly(dA) was annealed to 18-mer oligo(dT) modified with phosphate at the 5′-OH or 3′-OH at a 2:1 ratio as nucleotides. (**E**) Poly(dA) was annealed to 18-mer oligo(dT) with one (-C1), two (-C2), or four (-C4) additional dCs at the 3′-end at a 2:1 ratio as nucleotides. The same data with poly(dA)-oligo(dT) were plotted in graphs for the wild type (A–E) and for ^his^HLTF^ΔN^ (F, G) as controls. Error bars from at least two experiments are shown with the symbols. (**H**) DNA-binding assay. HLTF (upper panel) or ^his^HLTF^ΔN^ (middle panel) was incubated with M13mp18 ssDNA (ss) or dsDNA (ds) tethered with magnetic beads, or magnetic beads only (–), at 4°C for 2 min, and the beads were separated from the supernatants. Each fraction was analyzed by western blotting with an anti-HLTF antibody, and band intensities were measured. The relative values of binding fractions normalized by the amount of the input were plotted in a graph (bottom panel).

Determining the structure of DNA required for the stimulation of HLTF should provide information about where HLTF functions in cells. The quantification method was used to analyze the effects of various types of DNA on the chain-formation activity of HLTF. A titration experiment of poly(dA)-oligo(dT) showed that the chain-formation activity was stimulated in a dose-dependent manner (Figure 3A). Since poly(dA)-oligo(dT) is a 2:1 mixture of poly(dA) and 18-mer oligo(dT) as nucleotides, 150 pmol of poly(dA)-oligo(dT) contains 100 pmol of dA residues and 50 pmol of dT residues. To determine the specific poly(dA)-oligo(dT) structure required for the stimulation, poly(dA) and 18-mer oligo(dT) were examined individually (Figure 3A). Since poly(dA) has an exceptionally rigid structure mediated by strong base-stacking tendencies (51), we compared HLTF activity on poly(dT) with that on a typical and a generally more unstructured ssDNA (51). We also tested M13mp18 ssDNA, which consists of ssDNA regions and dsDNA regions generated by intramolecular annealing, and bacteriophage λ and M13mp18 RF I as dsDNAs (Figure 3B). The results indicated that poly(dA) or 18-mer oligo(dT) itself did not stimulate HLTF, dsDNA was inefficient, and unstructured ssDNA was better than dsDNA. The stimulation with M13mp18 ssDNA could be largely attributed to its unstructured ssDNA regions, in addition to the minor contribution of its dsDNA regions. However, they were all less efficient than poly(dA)-oligo(dT). These results suggested that a structure in which the oligonucleotide is annealed to the ssDNA, probably at the 3′- and/or 5′-end, is important. To test this possibility, the 3′- or 5′-end of the 18-mer oligo(dT) was blocked by modification with a biotin molecule or a phosphate. The results obtained with the modified oligonucleotides annealed with poly(dA), which are shown in Figure 3C and D, indicate that 3′-OH, but not 5′-OH, is required for the efficient stimulation of the chain-formation activity of HLTF.

Next, we examined whether the 3′-end had to be annealed to the template DNA to stimulate chain-formation activity, because stalled 3′-ends opposite damaged bases on the template do not always result in stable base pairing. To generate flaps at the 3′-end, one, two, or four dC residues were added to the 3′-end of the 18-mer oligo(dT). The modified oligonucleotides were annealed to poly(dA) and analyzed (Figure 3E). The results demonstrated that poly(dA)-oligo(dT) with a one-base flap was as effective as poly(dA)-oligo(dT) without flap bases for stimulating chain-formation activity, whereas poly(dA)-oligo(dT) with two- or four-base flap was less effective for stimulating chain-formation activity (Figure 3E). These results suggest that the key structure for the stimulation of the chain-formation activity is the primer end during DNA replication, which probably corresponds to a stalled 3′-end opposite damaged bases on the template strand.

### Different properties of the HIRAN-truncated mutant ^his^HLTF^ΔN^

Since the HIRAN-truncated mutant, ^his^HLTF^ΔN^, exhibited reduced chain-formation activity (Figure 2), the role of the HIRAN domain in the activity was investigated. Unlike wild-type HLTF, ^his^HLTF^ΔN^ was mostly stimulated by M13mp18 RF I, a dsDNA (Figure 3F), to a level comparable to that of the wild type stimulated by poly(dA)-oligo(dT). The effect of poly(dA)-oligo(dT) was considerably lower than that of the dsDNA (Figure 3F), and it was comparable to that of the wild type stimulated by poly(dA) annealed with 3′-modified oligo(dT) with biotin or phosphate (Figure 3C and D). In contrast to the wild type, poly(dT) was less efficient than M13mp18 ssDNA for stimulation, despite the fact that M13mp18 ssDNA stimulated ^his^HLTF^ΔN^ as well as wild-type HLTF (Figure 3F). This suggested that stimulation with M13mp18 ssDNA differed between wild-type HLTF and ^his^HLTF^ΔN^. Since ^his^HLTF^ΔN^ was well stimulated by dsDNA, stimulation with M13mp18 ssDNA might be due to its dsDNA regions, in addition to a minor contribution of unstructured ssDNA regions. These results suggested that the HIRAN domain was required for the 3′-OH-dependent stimulation. Consistently, the effect of the 3′ modification of oligo(dT) with biotin was marginal in the ^his^HLTF^ΔN^ mutant (Figure 3G).

To examine the DNA-binding property of HLTF directly, M13mp18 ss or dsDNA was attached to magnetic beads, and a pull-down assay was performed (Figure 3H). As shown in Figure 3H, HLTF and ^his^HLTF^ΔN^ bound to M13mp18 ssDNA with equivalent affinities, consistent with the results of ligase assays in which they were equally stimulated by M13mp18 ssDNA (Figure 3B and F). By contrast, HLTF had a lower affinity for M13mp18 dsDNA, whereas ^his^HLTF^ΔN^ showed a 3-fold higher affinity than HLTF (Figure 3H, bottom panel). These results revealed several properties of the catalytic and HIRAN domains. The catalytic activity of HLTF is intrinsically stimulated to the highest degree by dsDNA. The HIRAN domain prevents direct loading of the catalytic domain onto dsDNA, whereas it helps in recruiting the catalytic domain to dsDNA via the 3′-OH of primer termini. However, the mechanism underlying the HIRAN domain-dependent enhancement of the stimulation with unstructured ssDNA, as shown with poly(dT) (Figure 3B and F), remains unclear. The weak interaction between ssDNA and the HIRAN domain (9,10) might be involved in the enhancement.

### RFC and PCNA suppress the chain-formation activity of HLTF at the primer end

**Figure 4.**
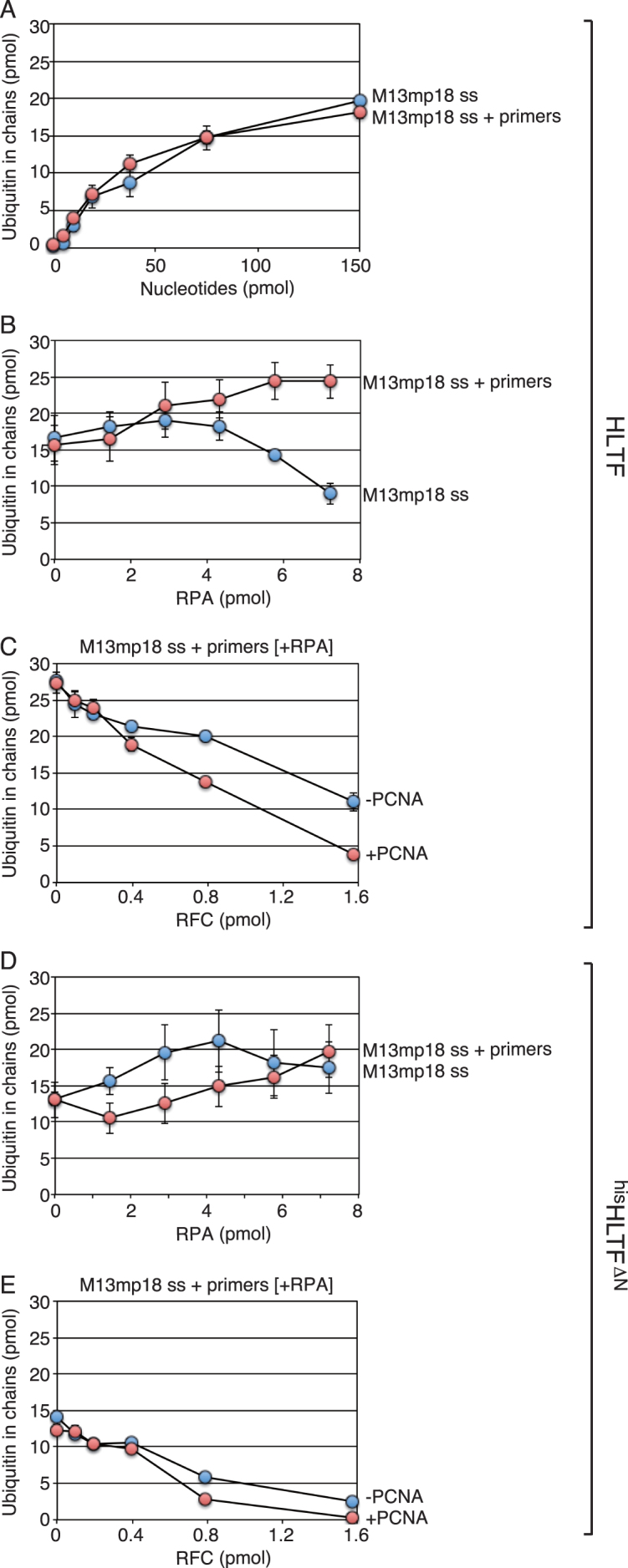
Chain-formation activity of HLTF with the multiply primed M13mp18 ssDNA and the indicated replication factors. The chain-formation activities of wild-type HLTF (**A–C**) and ^his^HLTF^ΔN^ (**D, E**) were analyzed under standard assay conditions containing E1, MMS2-UBC13, and ubiquitin at 30°C for 10 min with the indicated DNA and replication factors. The total amounts of ubiquitin in chains in each 25 μl reaction mixture were plotted. (A) Titrations of the multiply primed and not-primed M13mp18 ssDNA. The amounts of nucleotides correspond to those of the M13mp18 ssDNA backbone. (B, D) Titration of RPA with the indicated DNA (150 pmol nucleotides of the M13mp18 ssDNA backbone). (C, E) Titration of RFC with multiply primed M13mp18 ssDNA (150 pmol nucleotides of the M13mp18 ssDNA backbone) and RPA (7.3 pmol) in the absence or presence of PCNA (1 pmol). Error bars of at least two experiments are shown with symbols.

Because HLTF is apparently recruited to DNA structures of stalled primer termini, replication factors present around the primer termini during DNA replication might influence the ligase activity of HLTF. To examine the interaction between HLTF and replication factors present around the primer termini, 20 oligonucleotides (Supplementary Table S1) were annealed to M13mp18 ssDNA, generating a multiply primed M13mp18 ssDNA that was used for subsequent assays. Stimulation of HLTF by the multiply primed M13mp18 ssDNA was comparable to that induced by M13mp18 ssDNA itself (Figure 4A). The impact of the 3′-OH may have been masked by the effects of unstructured ssDNA regions of M13mp18 ssDNA. However, introduction of RPA had a stimulatory effect with the multiply primed M13mp18 ssDNA, whereas it had an inhibitory effect with M13mp18 ssDNA itself (Figure 4B), suggesting the specific stimulation of HLTF at the 3′-OH in the presence of RPA. By contrast, such properties were not observed in ^his^HLTF^ΔN^ (Figure 4D). A possible explanation is that ^his^HLTF^ΔN^ was activated by the remaining dsDNA regions generated by intramolecular annealing of M13mp18 ssDNA, even in the presence of RPA, and dsDNA regions of the multiply primed M13mp18 ssDNA (Figure 4D), since ^his^HLTF^ΔN^ is accessible to dsDNA in a manner independent of the 3′-OH (Figure 3). When RFC was introduced into this assay system with the RPA-coated multiply primed M13mp18 ssDNA in the presence or absence of PCNA, it strongly inhibited the chain-formation activity of HLTF, and PCNA enhanced the inhibition (Figure 4C). In the case of ^his^HLTF^ΔN^, the chain-formation activity was also inhibited in the presence of RFC and slightly enhanced by PCNA (Figure 4E). This result was unexpected because the chain-formation activity stimulated by the dsDNA regions generated by intramolecular annealing of M13mp18 ssDNA (Figure 4D) should be insensitive to RFC and PCNA because of the lack of 3′-OH ends, which is required for RFC binding. Therefore, this result suggested that such dsDNA regions are missing in the RPA-coated multiply primed M13mp18 ssDNA. Both binding of 20 primers and RPA may have efficiently disrupted the secondary structure of M13mp18 ssDNA, and the chain-formation activity stimulated with RPA-coated multiply primed M13mp18 ssDNA (Figure 4E) could be attributed to only the dsDNA regions generated with annealed primers.

**Figure 5.**
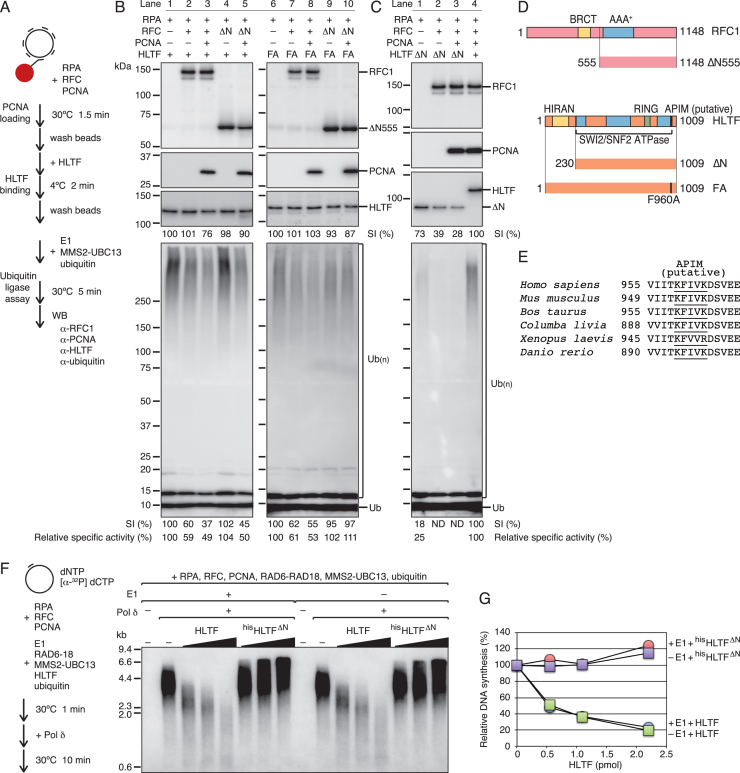
Suppression of the chain-formation activity of HLTF by interaction with RFC and PCNA. (**A**) Schematic of the experiments. Proteins were sequentially assembled on multiply primed M13mp18 ssDNA tethered to magnetic beads, and ubiquitin ligase assays were performed under standard assay conditions with the protein-bound DNA on the magnetic beads. (B, C) Western blot analysis of the assembled proteins, and ubiquitin chains generated by DNA-bound HLTF (**B**) and ^his^HLTF^ΔN^ (**C**) using anti-RFC1 (upper panels), anti-PCNA (second panels), anti-HLTF (third panels), and anti-ubiquitin antibodies (bottom panels). ‘–’ represents omitted proteins. ‘ΔN’ in RFC represents a mutant RFC consisting of ΔN555 RFC1. ‘FA’ represents the ^his^HLTF^FA^ mutant. ‘ΔN’ in HLTF represents ^his^HLTF^ΔN^. Each signal intensity (SI) (%) under the HLTF blotting panels in (B) and (C) indicates the relative intensity of HLTF signals after normalization as shown, and that under the ubiquitin blotting panels in (B) and (C) indicates the relative intensity of signals in each plot larger than 60 kDa after normalization as shown. ND, not determined because signal levels were indistinguishable from the background. Relative specific activity (%) was calculated as [SI (%) of ubiquitin blot]/[SI (%) of HLTF blot] × 100. (**D**) Schematic representation of the structures of RFC1 and HLTF and their mutants. (**E**) Alignment of putative APIM sequences of HLTF homologues. The accession numbers of the sequences were NP_001305864 (*H. sapiens*), NP_033236 (*M. musculus*), NP_001179215 (*B. taurus*), XP_005510651 (*C. livia*), XP_018117635 (*X. laevis*), and XP_005163433 (*D. rerio*). (**F**) Effects of HLTF and ^his^HLTF^ΔN^ on singly primed ss M13mp18 DNA replication with pol δ. Reaction mixtures containing RPA, RFC, PCNA, RAD6-RAD18, MMS2-UBC13, and ubiquitin in the presence or absence of E1 and HLTF as indicated, but lacking pol δ, were preincubated at 30°C for 1 min, and DNA synthesis was started by addition of pol δ. Reactions were performed at 30°C for 10 min. The amounts of HLTF and ^his^HLTF^ΔN^ were increased in the order of 0.55, 1.1, and 2.2 pmol. The reaction products were analyzed by 0.7% alkaline-agarose gel electrophoresis. ‘–’ indicates omitted proteins. (**G**) The radioactivity of [α-^32^P]dCMP incorporated into DNA was measured and normalized to the levels without HLTF.

Next, the molecular mechanisms underlying the inhibitory effect of RFC shown in Figure 4C were examined using the RPA-coated multiply primed M13mp18 ssDNA attached to magnetic beads where proteins were assembled (Figure 5A). First, PCNA was loaded by RFC to the DNA molecules on the magnetic beads in the presence of RPA. The beads were washed to remove unbound proteins and incubated with HLTF. Unbound HLTF was washed away, and the chain-formation activity of the DNA-bound HLTF was measured by introduction of the E1 MMS2-UBC13 and ubiquitin. The bound proteins and reaction products were analyzed by western blotting. As shown in Figure 5B, equivalent amounts of RFC in the presence or absence of PCNA were detected in the bound fraction, and HLTF was just slightly reduced in the presence of RFC and PCNA (lanes 1–3). Under these conditions, the chain-formation activity of HLTF was suppressed in the presence of RFC and further by PCNA (lanes 1–3), as demonstrated by decreased specific activity (described at the bottom of Figure 5B). This result reproduced the results of the solution assays (Figure 4C), and suggested that RFC and PCNA suppressed the chain-formation activity of HLTF on DNA, implying that HLTF specifically interacts with both RFC and PCNA. This is consistent with reports that HLTF associates with the replication fork (10), and RFC and HLTF are detected as components of a large complex isolated from human cells (52–54). The present co-immunoprecipitation assay detected the interaction in UV-irradiated cells (Supplementary Figure S2).

To examine whether interactions between HLTF and RFC are involved in the inhibition, the mutant RFC complex RFC^ΔN555^, consisting of the N-terminally truncated RFC1, ΔN555 (55), was tested (Figure 5B and D). RFC^ΔN555^ similarly loaded PCNA onto DNA and permitted HLTF binding (Figure 5B, lanes 1 and 4–5). However, the results of the ubiquitin assay showed that RFC^ΔN555^ was incapable of suppressing the chain-formation activity of HLTF (lane 4), which was recovered in the presence of PCNA (lane 5). These results supported that the N-terminal half of RFC1 is responsible for the suppression probably via interaction with HLTF.

Next, we analyzed the molecular mechanism underlying the suppression of HLTF activity by PCNA. We found that HLTF has a putative AlkB homologue 2 PCNA-interacting motif (APIM) (56) in its C-terminal region (Figure 5D and E). To examine the involvement of the putative APIM in the suppression of HLTF activity, the F960A mutant was purified as a histidine-tagged protein, ^his^HLTF^FA^ (Supplementary Figure S1B). Note that the chain-formation activity and ATPase activities of ^his^HLTF^FA^ were 3–4-fold and ∼2-fold lower than those of the wild type, respectively (Supplementary Figure S3). Since the F960 residue overlaps with the SWI2/SNF2 ATPase domain (Figure 5D), the reduction in the chain-formation activity could be caused by malfunction of the ATPase, at least in part (29,57); alternatively, as suggested in a previous report (58), the F960A mutation could be independently responsible for reductions in both activities. However, several HLTF ATPase mutants showed that reduced chain-formation activity correlated with reduced ATPase activity, supporting the former possibility, although ATPase activity was not absolutely required for chain-formation activity (58) (Supplementary Figure S3). When ^his^HLTF^FA^ was assembled on the beads, the chain-formation activity of ^his^HLTF^FA^ was suppressed by wild-type RFC (Figure 5B, compare lanes 6 and 7) but not by RFC^ΔN555^ (compare lanes 6 and 9) even in the presence of PCNA (lanes 6 and 9–10), suggesting that the assumed APIM-PCNA interaction was involved in the PCNA-mediated suppression.

The assay system was used to examine ^his^HLTF^ΔN^. The chain-formation activity of ^his^HLTF^ΔN^ isolated with the RPA-coated multiply primed M13mp18 ssDNA was considerably weaker than that of wild-type HLTF. Even in the absence of RFC, the activity was only 25% of that of HLTF under the suppressed condition in the presence of RFC and PCNA (Figure 5C, compare lanes 1 and 4), suggesting that the majority of the binding fraction of ^his^HLTF^ΔN^ was not productive in this assay. Nonetheless, the effect of RFC and PCNA on inhibiting the binding of ^his^HLTF^ΔN^ was determined (Figure 5C, lanes 1–3, and Supplementary Figure S4). RFC and PCNA occupied a certain space in the primer-mediated dsDNA region, which could compete with productive ^his^HLTF^ΔN^ binding. The remaining ^his^HLTF^ΔN^ in the presence of RFC or RFC and PCNA exhibited marginal chain-formation activity, suggesting that the RFC-resistant binding fraction was not productive. The results of the binding assays indicated that the assumed RFC-HLTF and PCNA-HLTF interactions did not play a major role in ternary complex formation. Indeed, such interactions were hardly detectable in pull-down assays (Supplementary Figure S5), supporting that the contacts between RFC-HLTF and HLTF-PCNA were weak, and although sufficient for suppression of the ligase activity, they did not support the formation of stable complexes. Thus, it will important to determine whether the putative APIM of HLTF actually functions as an APIM. Collectively, these results suggested that HLTF was loaded onto dsDNA via the primer ends by the HIRAN domain, and its chain-formation activity was suppressed by interactions with RFC and PCNA after loading at the primer end.

### HIRAN domain-dependent competition between HLTF and DNA pol δ in the *in vitro* replication system

To determine whether HLTF was accessible to the primer ends during DNA replication with DNA pol δ, DNA replication assays were performed with pol δ in the presence of ubiquitination enzymes and increasing amounts of HLTF (Figure 5F and G). DNA replication with pol δ is not influenced by the presence of RAD6-RAD18 or monoubiquitinated PCNA (43). HLTF associated with the primer ends in parallel with the dose-dependent inhibition of replication with pol δ (Figure 5F and G), indicating that HLTF possesses an intrinsic potential to associate primer termini by competition with DNA polymerases. The pattern of inhibition (Figure 5F) reflected a distributive action of HLTF (46), suggesting dynamic competition with repeating association-dissociation cycles between pol δ and HLTF. The association of HLTF was dependent on the HIRAN domain and independent of the ubiquitination of PCNA (Figure 5F and G). These results were consistent with the results of pull-down assays (Figure 5B and C), confirming that the interaction between the HIRAN domain and the 3′-OH plays a major role in complex formation at the primer ends.

### Polyubiquitination of PCNA in the complex with RFC and HLTF at the primer end

**Figure 6.**
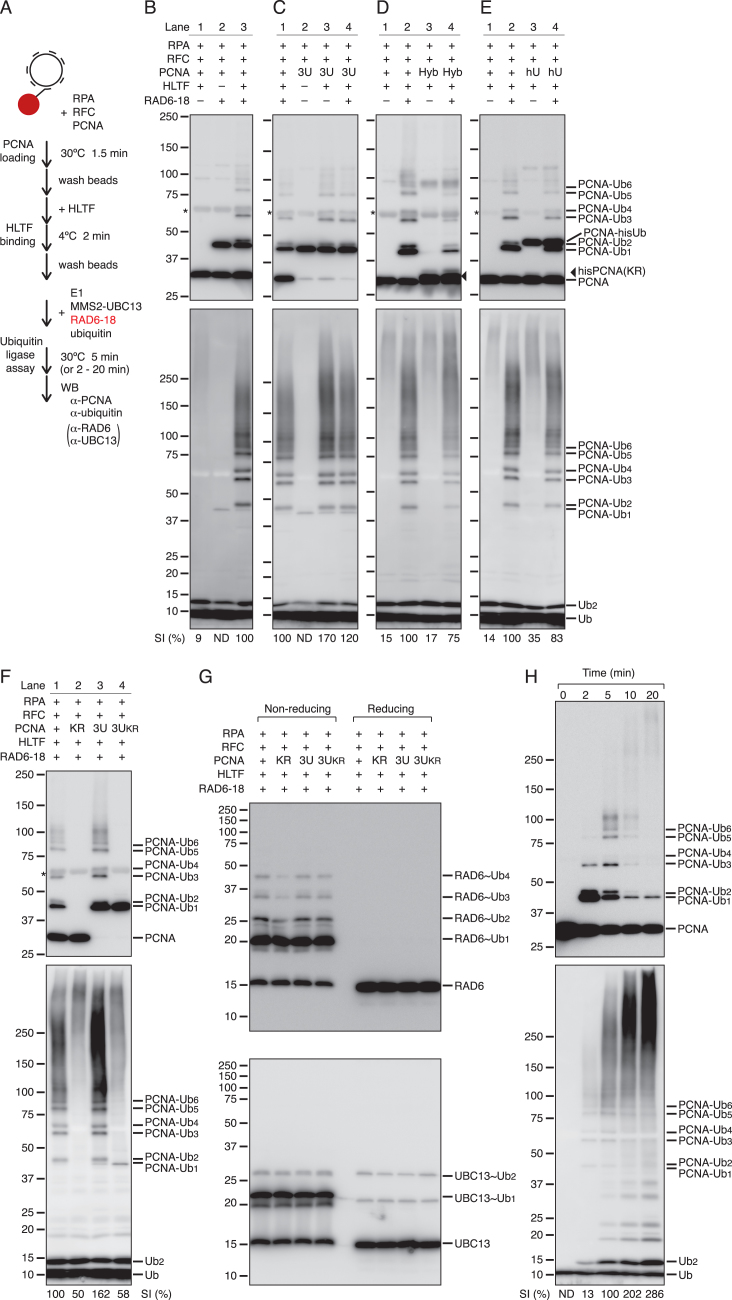
Two-step polyubiquitination of PCNA by sequential chain elongation in the RFC-PCNA-HLTF complex at the primer end. (**A**) Schematic of the experiments. This is identical to that of Figure 5A except for the addition of RAD6-RAD18 in the ubiquitin ligase assays. (**B–F**) Polyubiquitination of PCNA in the isolated complex. PCNA (upper panels) and the ubiquitin chains (bottom panels) were monitored by western blotting with anti-PCNA and anti-ubiquitin antibodies, respectively. ‘–’ represents omitted proteins; ‘3U’ represents three-subunit-monoubiquitinated PCNA; ‘Hyb’ represents a hybrid PCNA trimer consisting of ^his^PCNA^K164R^ and wild-type PCNA subunits. The position of ^his^PCNA^K164R^ is indicated by an arrowhead. To distinguish ^his^PCNA^K164R^ from unmodified PCNA, another image obtained by short exposure of (D, upper panel) is shown in Supplementary Figure S6. ‘hU’ represents partially monoubiquitinated PCNA with ^his^Ub (18); ‘KR’ represents the PCNA^K164R^ mutant; ‘3U_KR_’ represents three-subunit-monoubiquitinated PCNA with the K63R ubiquitin mutant; ‘*’ represents signals derived from cross reacted BSA. (**G**) Thiol-linked ubiquitin chains on E2s. Reaction products of (F) were analyzed by western blotting with anti-RAD6 (upper panel) or anti-UBC13 (lower panel) under non-reducing or reducing conditions. (**H**) Time course of polyubiquitination in the isolated complex. Each signal intensity (SI) (%) under ubiquitin blotting panels (B–F and H) indicates the relative intensity of signals in each plot larger than 37 kDa after normalization as shown. ND, not determined.

To examine whether HLTF in which chain-formation activity was suppressed could polyubiquitinate PCNA, the RAD6-RAD18 complex was introduced into the ubiquitination reaction on protein-bound DNA beads (Figure 6A). As shown in Figure 6B, the introduction of RAD6-RAD18 resulted in the mono- and polyubiquitination of PCNA on DNA in the absence (lane 2) and presence (lane 3) of HLTF, respectively. Since a commonly used anti-ubiquitin monoclonal antibody, P4D1, is poor at detecting monoubiquitinated PCNA and ubiquitin monomers, probably because of its lower affinity for the single ubiquitin moiety and free ubiquitin, the ubiquitinated PCNA molecules were also visualized with anti-PCNA antibody. The results indicated that, even when the chain-formation activity was suppressed, HLTF in the complex was active in the polyubiquitination of PCNA. Therefore, the mode of polyubiquitination under these conditions was examined. First, we examined whether monoubiquitinated PCNA can be a substrate under these conditions. Three-subunit-monoubiquitinated PCNA was used instead of unmodified PCNA (Figure 6C). The results showed that monoubiquitinated PCNA was polyubiquitinated in the absence of RAD6-RAD18 (Figure 6C), indicating a sequential reaction from monoubiquitinated PCNA to polyubiquitinated PCNA, as previously demonstrated in yeast RAD5 (16,17).

We previously provided biochemical evidence that monoubiquitinated PCNA is a poor substrate for polyubiquitination by HLTF (18). In those experiments, we used partially monoubiquitinated PCNA as the substrate. However, it remained unclear why the monoubiquitinated subunits of the partially monoubiquitinated PCNA trimer are not targeted for the ligase reaction by HLTF. One possible explanation is that a subunit of the PCNA trimer cannot be polyubiquitinated until all three subunits are monoubiquitinated. Another possibility is that, when a subunit of the PCNA trimer that interacts with RFC and HLTF at the primer end is monoubiquitinated by RAD6-RAD18, only the resulting ubiquitin moiety can act as the substrate of HLTF. To examine these possibilities, we prepared hybrid trimers consisting of histidine-tagged PCNA^K164R^ (^his^PCNA^K164R^) and untagged wild-type PCNA (Supplementary Figure S1C). Since hybrid PCNA was purified via Ni^2+^ affinity binding, at least one subunit in the trimer is the K164R mutant, and the three subunits are therefore not simultaneously ubiquitinated. The property of the hybrid PCNA in the reaction with M13mp18 ssDNA tethered on beads was examined next (Figure 6D). The results showed that hybrid PCNA could be polyubiquitinated when it was monoubiquitinated by RAD6-RAD18 (Figure 6D, lane 4; see also Supplementary Figure S6). Next, we examined whether partially monoubiquitinated PCNA in the isolated complex could be polyubiquitinated. The ubiquitin moieties of partially monoubiquitinated PCNA were histidine-tagged to distinguish them from unmodified subunits ubiquitinated during the reactions (see Supplementary Figure S7). The results showed that partially monoubiquitinated PCNA itself could not be polyubiquitinated (Figure 6E, lane 3). In the presence of RAD6-RAD18, polyubiquitination of PCNA was detected (Figure 6E, lane 4), although the ^his^Ub moiety was hardly polyubiquitinated, as shown by the migration of polyubiquitinated PCNA signals in a manner similar to that of unmodified subunits but not ^his^Ub-modified subunits (Figure 6E, lane 4, and see also Supplementary Figure S7). The inefficiency of HLTF polyubiquitination of partially monoubiquitinated PCNA cannot be attributed to the histidine-tag of ^his^Ub, because three-subunit-monoubiquitinated PCNA with ^his^Ub was well polyubiquitinated (Supplementary Figure S7). These results indicated that a mechanism couples the monoubiquitination of PCNA to its polyubiquitination in the complex containing RFC, PCNA, and HLTF at the primer ends.

### Evidence of sequential chain elongation in the complex at the primer end

Since we identified a reaction condition in which monoubiquitinated PCNA behaves as the substrate for polyubiquitination, as previously shown in yeast RAD5 (16,17), we next examined the mode of polyubiquitination in this condition, namely, whether a preformed ubiquitin chain is transferred to monoubiquitinated PCNA in one reaction (*en bloc* transfer) or the ubiquitin chain is elongated step-by-step. To this end, two criteria for the *en bloc* transfer reaction were tested (18). One is the accumulation of the thiol-linked ubiquitin chains on RAD6 when subsequent chain transfer is prevented. The other is constant ubiquitin chain length as a function of time (18). First, the accumulation of thiol-linked ubiquitin chains on E2s was examined. As controls for blocked ubiquitination, the PCNA mutant PCNA^K164R^ and three-subunit-monoubiquitinated PCNA with the ubiquitin mutant Ub^K63R^ were prepared (18). As shown in Figure 6F, subsequent ubiquitination was not observed in these mutants. Under these conditions, thiol-linked ubiquitin chains on RAD6 and UBC13 were analyzed (Figure 6G). The results showed that the chains on E2s were shorter than that detected on PCNA, and chain accumulation was not detected, suggesting that these chains are not direct intermediates for the polyubiquitination of PCNA, but rather secondary products generated in solution out of the complex. Next, the time course of the reaction was examined in the isolated complex. As shown Figure 6H, ubiquitin chains were elongated in a time-dependent manner. At 2 min, mono- and di-ubiquitinated PCNA were the predominant forms; the peak fraction was shifted to di- or tri-ubiquitinated forms at 5 min, and most of the monoubiquitinated PCNA was shifted to long polyubiquitinated forms at 10 min, followed by elongation to nearly gel top at 20 min. The majority of monoubiquitinated PCNA was converted to polyubiquitinated forms at 10 min. These results clearly indicated that polyubiquitination followed monoubiquitination with RAD6-RAD18 through step-by-step elongation.

### Regulation of the mode of polyubiquitination by RFC

**Figure 7.**
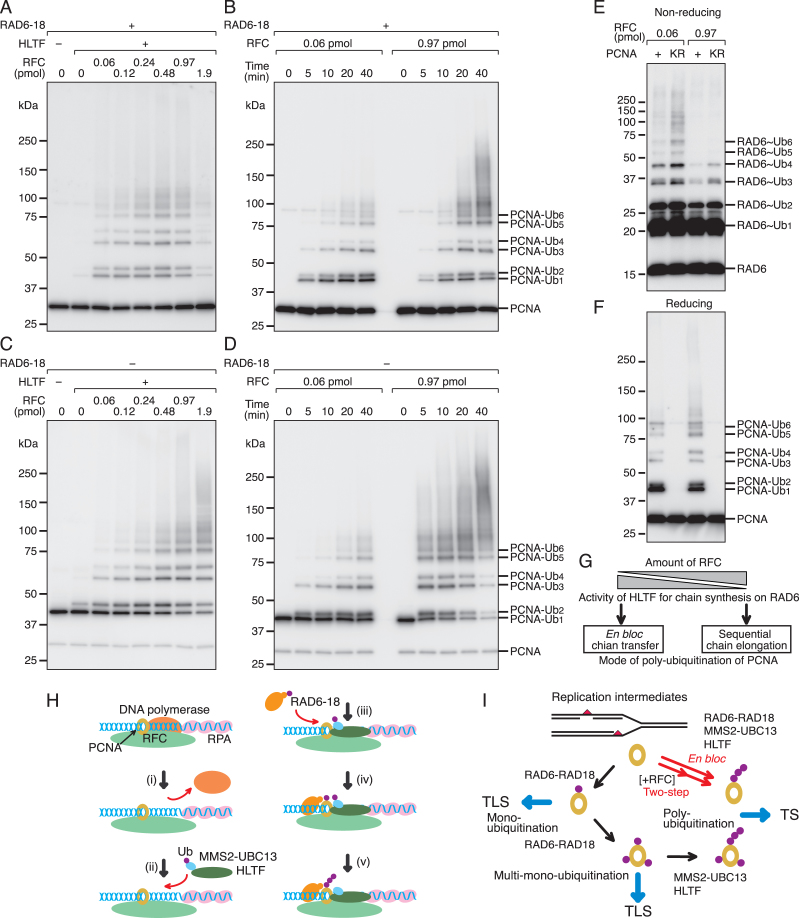
RFC regulates the mode of polyubiquitination. (A, C) Titration of RFC in the bulk reaction with PCNA in the presence of RAD6-RAD18 (**A**) or three-subunit-monoubiquitinated PCNA in the absence of RAD6-RAD18 (**C**). (B, D) Time course of the reactions with PCNA in the presence of RAD6-RAD18 (**B**) or three-subunit-monoubiquitinated PCNA in the absence of RAD6-RAD18 (**D**) under conditions with different amounts of RFC. Reactions were performed under standard assay conditions for the bulk reaction containing E1, MMS2-UBC13, ubiquitin, HLTF, the indicated PCNA, and the indicated amounts of RFC in the presence or absence of RAD6-RAD18 at 30°C for 10 min with 150 pmol of poly(dA)-oligo(dT) nucleotides. Reaction products were analyzed by western blotting with an anti-PCNA antibody. (**E**) Thiol-linked ubiquitin chains on RAD6. Reactions were performed under standard assay conditions for the bulk reaction containing E1, MMS2-UBC13, RAD6-RAD18, ubiquitin, HLTF, the indicated PCNA, and the indicated amounts of RFC at 30°C for 10 min with 150 pmol of poly(dA)-oligo(dT) nucleotides. Reaction products were analyzed by western blotting under non-reducing conditions with an anti-RAD6 antibody. (**F**) The products in (E) were analyzed by western blotting under reducing conditions with an anti-PCNA antibody. (**G**) Model of the regulatory mode of polyubiquitination of PCNA. RFC prevents ubiquitin chain synthesis on E2, restricting the reaction to step-by-step chain elongation of the ubiquitin moiety of ubiquitinated PCNA. (**H**) Model of the action of HLTF at stalled primer ends. (i) Dissociation of DNA polymerases. (ii) Recruitment of HLTF to the stalled primer end by the HIRAN domain. The ligase activity of HLTF for ubiquitin chain formation on E2 is suppressed by interaction with RFC and PCNA. (iii) Recruitment of RAD6-RAD18. (iv) RAD6-RAD18 monoubiquitinates PCNA interacting with HLTF. (v) HLTF polyubiquitinates the ubiquitin moiety of the resulting monoubiquitinated PCNA. (**I**) Damage tolerance pathways regulated by the ubiquitination of PCNA. Polyubiquitination of PCNA occurs via three biochemical reaction pathways. One is a coupling reaction of mono- and polyubiquitination via direct chain transfer from RAD6∼Ub_(n)_ to PCNA mediated by RAD18, as shown by a long red arrow (18). The second is a two-step coupling reaction mediated by RFC, as shown by two consecutive red arrows. The details of this pathway are shown in (H). The third is an uncoupling pathway, as shown by three short black arrows. After all subunits of PCNA are monoubiquitinated, the PCNA molecule is polyubiquitinated by HLTF. The three-subunit-monoubiquitinated PCNA promotes not only TLS but also the third pathway for damage tolerance (63).

The regulation of the mode of polyubiquitination was examined next. We previously demonstrated that the *en bloc* reaction was predominant in the bulk reaction (18), whereas step-by-step elongation was the predominant reaction in the isolated complex. The crucial difference is the amount of RFC. In the former, catalytically sufficient amounts of RFC for loading PCNA were introduced, whereas in the latter reaction, stoichiometric amounts of RFC were used to form a complex at the primer ends. To determine the effect of the amount of RFC, polyubiquitination of PCNA was re-examined in the bulk reaction. As shown in Figure 7A, polyubiquitination of PCNA was detected in the presence of RFC at 0.06–0.97 pmol. The reaction was inhibited in the presence of the highest amounts of RFC at 1.9 pmol. The time courses of the reactions were analyzed in the presence of lower (0.06 pmol) or higher (0.97 pmol) amounts of RFC (Figure 7B). With a low amount of RFC, the reactions consisted of *en bloc* transfer, because chain length was constant over time. By contrast, with higher amounts of RFC, chain length increased in a time-dependent manner (Figure 7B). The same experiments were performed with three-subunit-monoubiquitinated PCNA without RAD6-RAD18 (Figure 7C and D). The results showed that three-subunit-monoubiquitinated PCNA was also polyubiquitinated in the bulk reaction without RAD6-RAD18, and that this polyubiquitination was efficient in the presence of higher amounts of RFC (Figure 7C). The time courses demonstrated chain elongation with higher amounts of RFC, but not with lower amounts of RFC (Figure 7D). Partially monoubiquitinated PCNA was not polyubiquitinated without RAD6-RAD18 in the range of RFC examined (Supplementary Figure S8). Finally, the accumulation of ubiquitin chains on RAD6 was examined using the bulk reaction with wild-type PCNA or PCNA^K164R^ to prevent subsequent chain transfer. As shown in Figure 7E, ubiquitin chains on RAD6 were detected with lower amounts of RFC and the length of the chains was equivalent to that on PCNA (Figure 7F). When chain transfer was prevented with PCNA^K164R^, chain accumulation was detected on RAD6. By contrast, higher amounts of RFC inhibited chain formation on RAD6, and only short chains were detected on RAD6, confirming the step-by-step chain elongation on PCNA in the presence of higher amounts of RFC (Figure 7E). These results revealed a novel function of RFC in the regulation of HLTF, which determined the mode of polyubiquitination of PCNA (Figure 7G).

## DISCUSSION

HLTF, a human homologue of yeast RAD5, is a multi-functional protein consisting of multiple domains. In the present study, we found that the ubiquitin ligase activity of HLTF is intrinsically stimulated by dsDNA, and the HIRAN domain prevents direct loading of the catalytic domain on dsDNA by restricting its recruitment to the 3′-OH of primer termini. This is consistent with recent reports that the HIRAN domain recognizes the 3′-OH (9–11). In addition, we found that the recruitment of HLTF was not prevented by a one-base flap at the 3′-end. When replicative DNA polymerases stall opposite template damaged bases, extension often stops just before or after one base insertion against the damaged base. The function of HLTF at the 3′-end is well adapted to stalled primer ends, suggesting that HLTF has the potential to be recruited to the stalled primer ends after the dissociation of replicative DNA polymerases. This biochemical property of HLTF, namely, its accessibility to primer ends, was demonstrated by the competition assay with DNA pol δ. Since human pol δ spontaneously dissociates from the 3′-end during replication at least *in vitro* (46,59,60), the recruitment of HLTF was regarded as a dynamic competitive inhibition of DNA synthesis with pol δ. The association was dependent on the HIRAN domain of HLTF and independent from the ubiquitination of PCNA, suggesting that the interaction between HIRAN and the 3′-OH is the most important for the initial association with the stalled primer ends (Figure 7H). This is consistent with reports that yeast RAD5 forms subnuclear foci in S phase in a manner dependent on HIRAN but independent of the catalytic activities (29,61).

The most important finding of the present study is that the ubiquitin ligase activity of HLTF is regulated by RFC and PCNA, although we were unable to provide direct evidence of RFC-HLTF and PCNA-HLTF interactions. The significance of the regulation in the *in vivo* setting remains to be elucidated, as HLTF-mediated PCNA polyubiquitination is not detectable in available human cells. However, we are currently making every effort to establish an assay system for polyubiquitinated PCNA *in vivo*. Nonetheless, our biochemical data demonstrated three modes of polyubiquitination of PCNA (Figure 7I). One is RAD18-mediated *en bloc* chain transfer of the preformed ubiquitin chain by HLTF-MMS2-UBC13 on RAD6 (18), which is activated in the absence of RFC. Here, monoubiquitinated PCNA intermediates are not generated. The second is monoubiquitination-coupled two-step polyubiquitination at primer termini. The activity of HLTF for ubiquitin chain synthesis on RAD6 is suppressed by interactions with RFC and PCNA in the RFC-PCNA-HLTF complex at primer ends (Figure 7G and H). The N-terminal of the RFC1 subunit of RFC and the newly found putative APIM of HLTF are required for the suppression. PCNA in the complex can be a target for monoubiquitination by RAD6-RAD18, and the resultant ubiquitin moiety is elongated step-by-step by the ligase activity of HLTF (Figure 7H and I). The two-step reaction was well coupled, because most of the monoubiquitinated PCNA was converted to polyubiquitinated PCNA at 10 min. In addition, polyubiquitination only occurred on RAD6-RAD18-mediated *de novo* monoubiquitinated PCNA in a complex pre-formed with RFC and HLTF (Figure 7H and I). Thus, monoubiquitinated intermediates in the pre-formed RFC-PCNA-HLTF complex were hardly released from the complex. The third mode is polyubiquitination uncoupled from monoubiquitination. When PCNA was monoubiquitinated in the absence of HLTF, it was not polyubiquitinated by subsequently recruited HLTF unless all three-subunits of PCNA were monoubiquitinated, because polyubiquitination by HLTF can only occur when all three-subunits of PCNA are already monoubiquitinated (Figure 7I). Therefore, HLTF polyubiquitination can occur at distinct spatial and temporal locations from those of monoubiquitination. Since both *en bloc* ubiquitin chain transfer and step-by-step chain elongation were observed in yeast RAD5 (16–18), and the N-terminal HIRAN domain is conserved (8), we believe that the biochemical properties of RAD5 are similar to those of HLTF. This is supported by reports that RAD5 also interacts with PCNA (17,58,62).

The results of this study suggest a mechanism exists to select one DDT pathway at stalled primer ends. When PCNA is monoubiquitinated by RAD6-RAD18 before the association of HLTF/RAD5 with the stalled 3′-OH, the monoubiquitinated subunit is not polyubiquitinated until all three subunits are monoubiquitinated, thereby promoting only the TLS pathway. If DNA synthesis is not restored by TLS for a long time, the same PCNA molecule persists at the stalled primer end and then can be fully monoubiquitinated by RAD6-RAD18. The resultant three-subunit-monoubiquitinated PCNA molecule is now the substrate for polyubiquitination by HLTF/RAD5 (Figure 7I). This suggests that, if TLS fails, the DDT pathway can switch from TLS, dependent on RAD6-RAD18-mediated monoubiquitination of PCNA, to TS, dependent on HLTF-mediated polyubiquitination of the three-subunit-monoubiquitinated PCNA generated during TLS. The switch could be activated depending on the levels of monoubiquitination of PCNA generated by RAD6-RAD18 during TLS. Because mono- and polyubiquitination are not coupled in this case, polyubiquitination can occur at a later time point such as at G2 phase. The uncoupling reaction may reflect the *in vivo* situation reported in genetic studies, i.e. TS operates when TLS fails (24,25,38–40). On the other hand, when HLTF/RAD5 is recruited to the stalled primer end first, the polyubiquitination reaction is initiated by *de novo* monoubiquitination by RAD6-RAD18 (Figure 7H and I). In the absence of RFC, the recruited HLTF/RAD5 generates a ubiquitin chain on RAD6, and PCNA is then polyubiquitinated by the ligase activity of RAD18 (Figure 7I) (18). If these reactions initiated by recruitment of HLTF/RAD5 to stalled primer ends predominate under certain cellular conditions, TS would precede TLS (22,23,25,29,30). Further studies are needed to elucidate the regulation of the recruitment of HLTF/RAD5 to the stalled primer ends.

## Supplementary Material

Supplementary Data
