# Supplementary material for: Regulation of HLTF-mediated PCNA polyubiquitination by RFC and PCNA monoubiquitination levels determines choice of damage tolerance pathway
Source: Nucleic Acids Res. 2018 Oct 18;46(21):11340–56. doi: 10.1093/nar/gky943 (PMC6265450; doi:10.1093/nar/gky943)
Supplement: Supplementary Data [file gky943_supplemental_files.pdf]

Supplementary Table S1

Synthetic oligonucleotides for multiply primed M13mp18 single-stranded DNA

Oligonucleotide	Sequence
M13-30	AAGAATTAGCAAAATTAAGCAATAAAGCCTCAGAGC
M13-370	GAACGGTAATCGTAAAACTAGCATGTCAATCATATG
M13-710	AGTTTGAGGGGACGACGACCGTATCGGCCTCAGGAA
M13-1260	TTTCTTTTCACCAGCGAGACGGGCAACAGCTGATTG
M13-1600	AGGGAGCCCCCGATTTAGAGCTTGACGGGGAAAGCC
M13-1940	AACCGTTGTAGCAATACTTCTTTGATTAGTAATAAC
M13-2280	CCATTAAAAATACCGAACGAACCACCAGCAGAAGAT
M13-2620	TAATTTTAAAAGTTTGAGTAACATTATCATTTTGCG
M13-2960	AATTACCTGAGCAAAAGAAGATGATGAAACAAACAT
M13-3300	AGAAAACTTTTTCAAATATATTTTAGTTAATTTCAT
M13-3640	AATGCAGAACGCGCCTGTTTATCAACAATAGATAAG
M13-3980	TTTTATCCTGAATCTTACCAACGCTAACGAGCGTCT
M13-4320	GCAGATAGCCGAACAAAGTTACCAGAAGGAAACCGA
M13-4660	GCAAAATCACCAGTAGCACCATTACCATTAGCAAGG
M13-5000	ATTGGCCTTGATATTCACAAACGAATGGATCTTCAT
M13-5340	ACCGTACTCAGGAGGTTTAGTACCGCCACCCTCAGA
M13-5680	ATCTCCAAAAAAAAAGGCTCCAAAAGGAGCCTTTAA
M13-6020	GCGAAAGAATACACTAAAACACTCATCTTTGACCCC
M13-6360	ACGAGTAGTAAATTGGGCTTGAGATGGTTTAATTTC
M13-6700	TAAAATGTTTAGACTGGATAGCGTCCAATACTGCGG

Supplementary Figure Legends

Supplementary Figure S1. The indicated proteins (500 ng, **A**; and 300 ng, **B**), and 500 ng of hybrid PCNA consisting of the wild-type PCNA and $^{his}PCNA^{K164R}$ subunits (**C**), were analyzed by SDS-PAGE with Coomassie Brilliant Blue (CBB) staining. The ratio of PCNA to $^{his}PCNA^{K164R}$ was 1:1.7 as determined by scanning the wet gel. FA, F960A mutant; KR, K300R mutant; DEAA, D557A/E558A double mutant.

Supplementary Figure S2. Analysis of the RFC-HLTF interaction *in vivo*. $HLTF^{-/-}/^{3xFLAG}HLTF$ and $HLTF^{-/-}$/vector cells were irradiated with UVC (15 J/m^2). After 3 h of incubation, chromatin fractions were prepared and subjected to immunoprecipitation (IP) with anti-FLAG agarose. Fractions eluted with the 3×FLAG peptide were analyzed by western blotting with the indicated antibodies. An asterisk represents a cross-reacted signal with anti-FLAG antibody. To determine the IP efficiency for RFC, the IP signal intensities of RFC bands (relative RFC) (top panel) were divided by ^{3xFLAG}HLTF signals (relative HLTF) (middle panel).

Supplementary Figure S3. Enzyme activities of mutant HLTF. (**A**) Chain-formation activity. Reactions were performed under standard assay conditions containing E1, MMS2-UBC13, and ubiquitin at 30°C for 10 min with 150 pmol of poly(dA)-oligo(dT) nucleotides and increasing amounts of enzymes at 30°C for 10 min. The total amounts of ubiquitin in chains in a 25 μL reaction mixture were plotted. (**B**) ATPase activity. Reactions were performed under standard assay conditions with 150 pmol of poly(dA)-oligo(dT) nucleotides and increasing amounts of enzymes at 30°C for 90 min. E1, MMS2-UBC13, and ubiquitin were not included in the assays. The total amounts of hydrolyzed ATP in a 25 μL reaction mixture were plotted. FA, F960A mutant; KR, K300R mutant; DEAA, D557A/E558A double mutant.

Supplementary Figure S4. Pull-down experiment of $^{his}HLTF^{\Delta N}$ on multiply primed M13mp18 ssDNA tethered to magnetic beads, related to Figure 5C (third panel). The reactions were performed as described in Figure 5A. The unbound fractions were obtained after 2 min of incubation with HLTF. Equivalent amounts of bound and unbound fractions were loaded on the gel. In Figure 5C (third panel), only the plot of the bound fraction is

shown. '–' indicates omitted proteins; 'ΔN' indicates ^{his}HLTF$^{\Delta N}$.

Supplementary Figure S5. Analysis of the RFC-HLTF and PCNA-HLTF interactions. (**A, C**) Pull-down assay of PCNA (**A**) or RFC (**C**) with histidine-tagged proteins. The indicated proteins or buffer control (-) immobilized on Profinity™ IMAC Ni-Charged Resin was incubated with PCNA (**A**) or RFC (**C**). After washing the beads, bound proteins were analyzed by western blotting. PCNA and RFC1 were detected using anti-PCNA (**A**) and anti-RFC1 (**C**) antibodies, respectively (upper panels). Pol η was the positive control for binding to PCNA (**A**). The membranes were stained with CBB (bottom panels). (**B, D**) Pull-down assay of PCNA (**B**) or HLTF (**D**) with the indicated GST-fusion proteins. The indicated proteins immobilized on Glutathione Sepharose™ were incubated with PCNA (**B**) or ^{his}HLTF (**D**). After washing the beads, bound proteins were eluted with a buffer containing 10 mM glutathione and analyzed by western blotting. PCNA (**B**) and ^{his}HLTF (**D**) were detected using anti-PCNA and anti-HLTF antibodies, respectively (upper panels). GST-pol η (557-713) was the positive control for binding to PCNA (**B**). The membranes were stained with CBB (bottom panels).

Supplementary Figure S6. Polyubiquitination of the hybrid PCNA in the complex at the primer end, related to Figure 6D, upper panel. The image was obtained by a short exposure of the membrane shown in Figure 6D, upper panel.

Supplementary Figure S7. Polyubiquitination of the three-subunit-monoubiquitinated PCNA with histidine-tagged ubiquitin in the isolated complex, related to Figure 6E. The experiment was performed as described in Figure 6A. '–' represents omitted proteins; '3U' represents three-subunit-monoubiquitinated PCNA; '3hU' represents three-subunit-monoubiquitinated PCNA with histidine-tagged ubiquitin.

Supplementary Figure S8. Partially monoubiquitinated PCNA is hardly polyubiquitinated in the absence of RAD6-RAD18. (**A**) Titration of RFC in the bulk reaction with partially monoubiquitinated PCNA with histidine-tagged ubiquitin in the absence of RAD6-RAD18. Reactions were performed under standard assay conditions for the bulk reaction containing E1, MMS2-UBC13, ubiquitin, HLTF, partially monoubiquitinated PCNA with histidine-tagged ubiquitin, and the indicated amounts of RFC with 150 pmol of poly(dA)-oligo(dT)

nucleotides at 30°C for 10 min. (**B**) Time course of the reactions with partially monoubiquitinated PCNA with histidine-tagged ubiquitin in the absence of RAD6-RAD18 and in the presence of the indicated amounts of RFC. (**C**) Comparison of the reaction efficiency under different conditions. Reactions were performed under standard assay conditions for the bulk reaction containing E1, MMS2-UBC13, ubiquitin, HLTF, the indicated PCNA, and the indicated amounts of RFC with 150 pmol of poly(dA)-oligo(dT) nucleotides in the presence or absence of RAD6-RAD18 at 30°C for 0 or 40 min. The reaction products were analyzed by western blotting with an anti-PCNA antibody. '–' represents omitted proteins; '3U' represents three-subunit-monoubiquitinated PCNA; 'hU' represents partially monoubiquitinated PCNA with histidine-tagged ubiquitin.

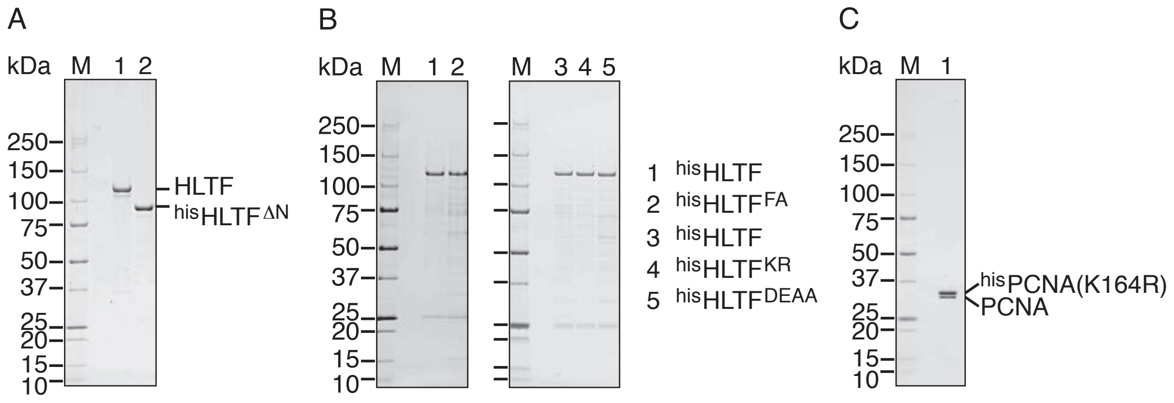

Figure S1

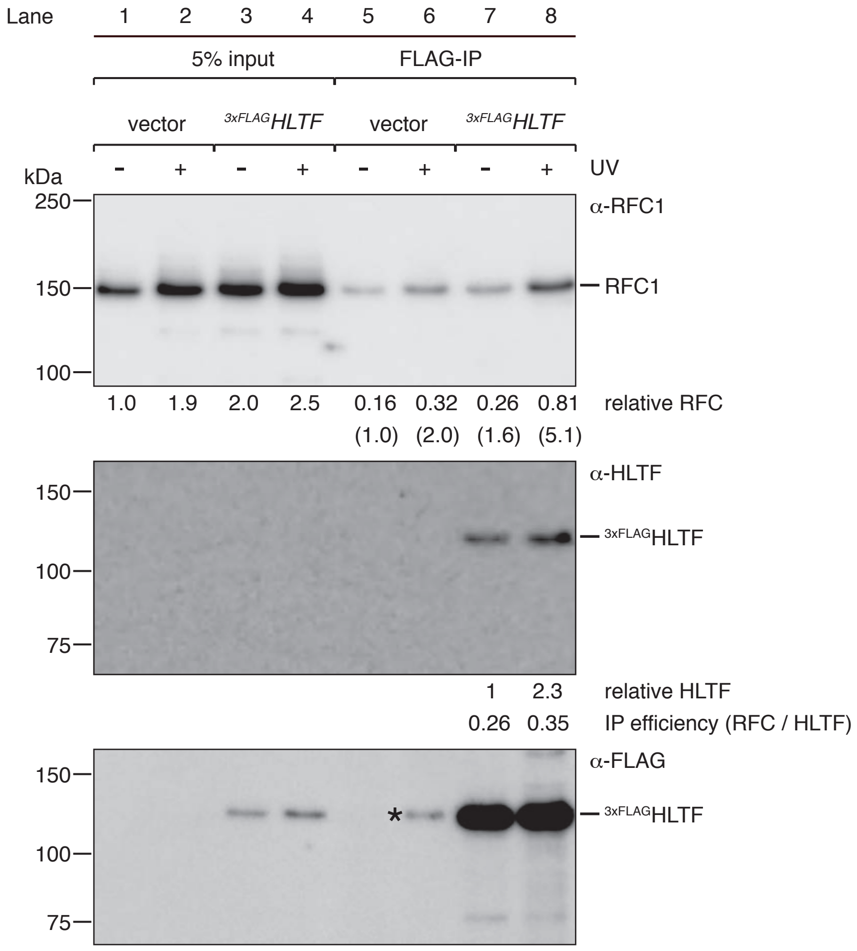

Figure S2

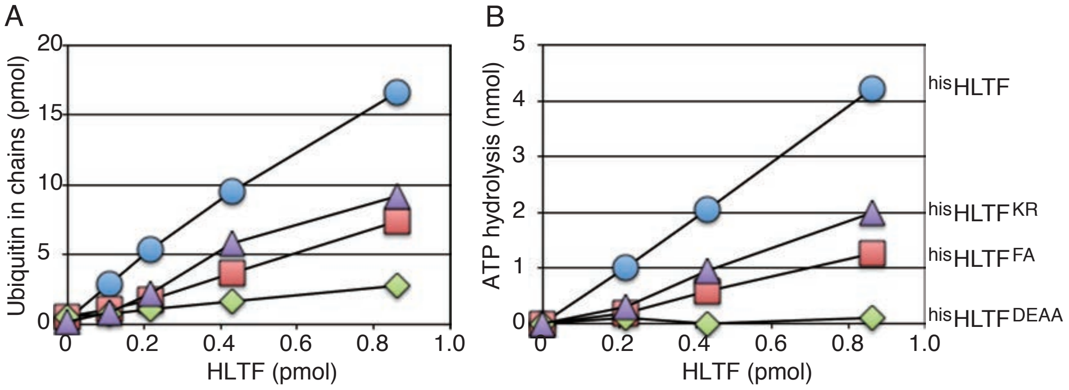

Figure S3

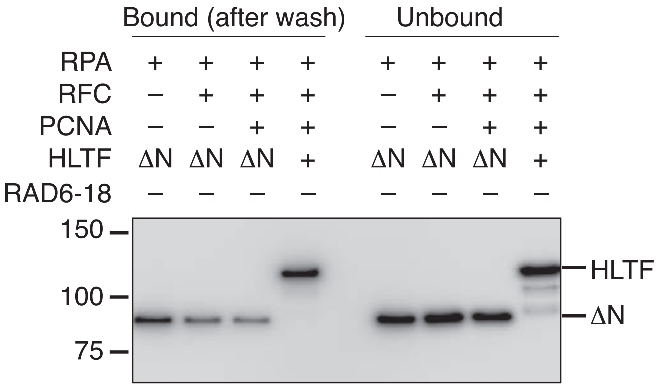

Figure S4

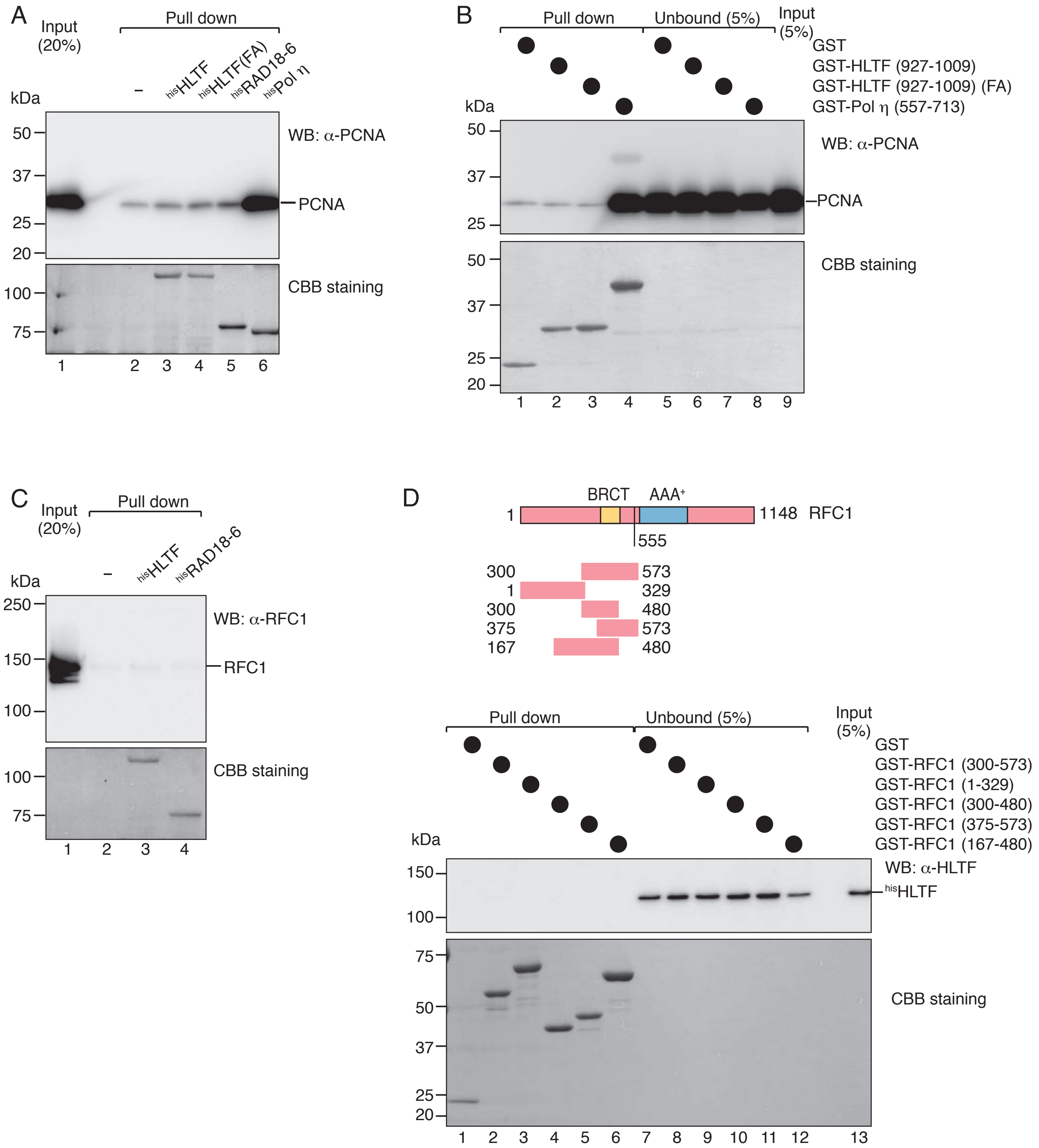

A
Input (20%)
Pull down
kDa
–
hisHLTF
hisHLTF(FA)
hisRAD18-6
hisPol η
WB: α-PCNA
PCNA
CBB staining
B
Pull down
Unbound (5%)
Input (5%)
GST
GST-HLTF (927-1009)
GST-HLTF (927-1009) (FA)
GST-Pol η (557-713)
C
WB: α-RFC1
RFC1
D
BRCT
AAA+
1148 RFC1
555
WB: α-HLTF
hisHLTF
GST-RFC1 (300-573)
GST-RFC1 (1-329)
GST-RFC1 (300-480)
GST-RFC1 (375-573)
GST-RFC1 (167-480)

Figure S5

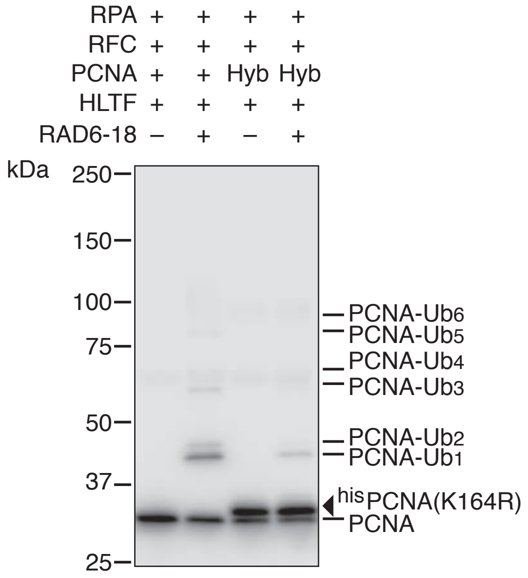

Figure S6

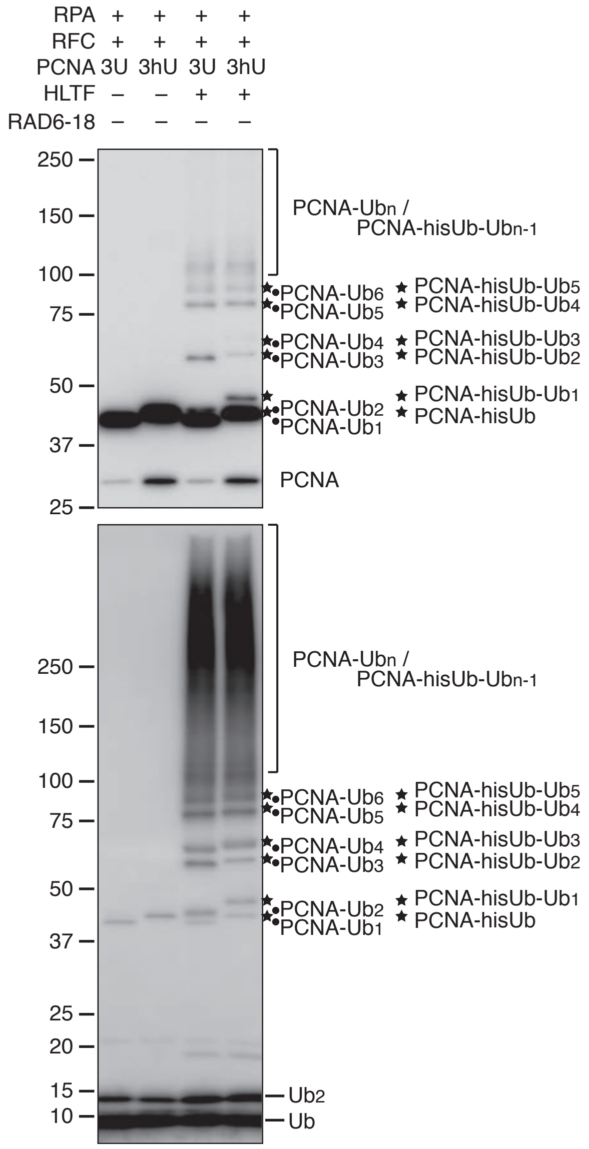

Figure S7

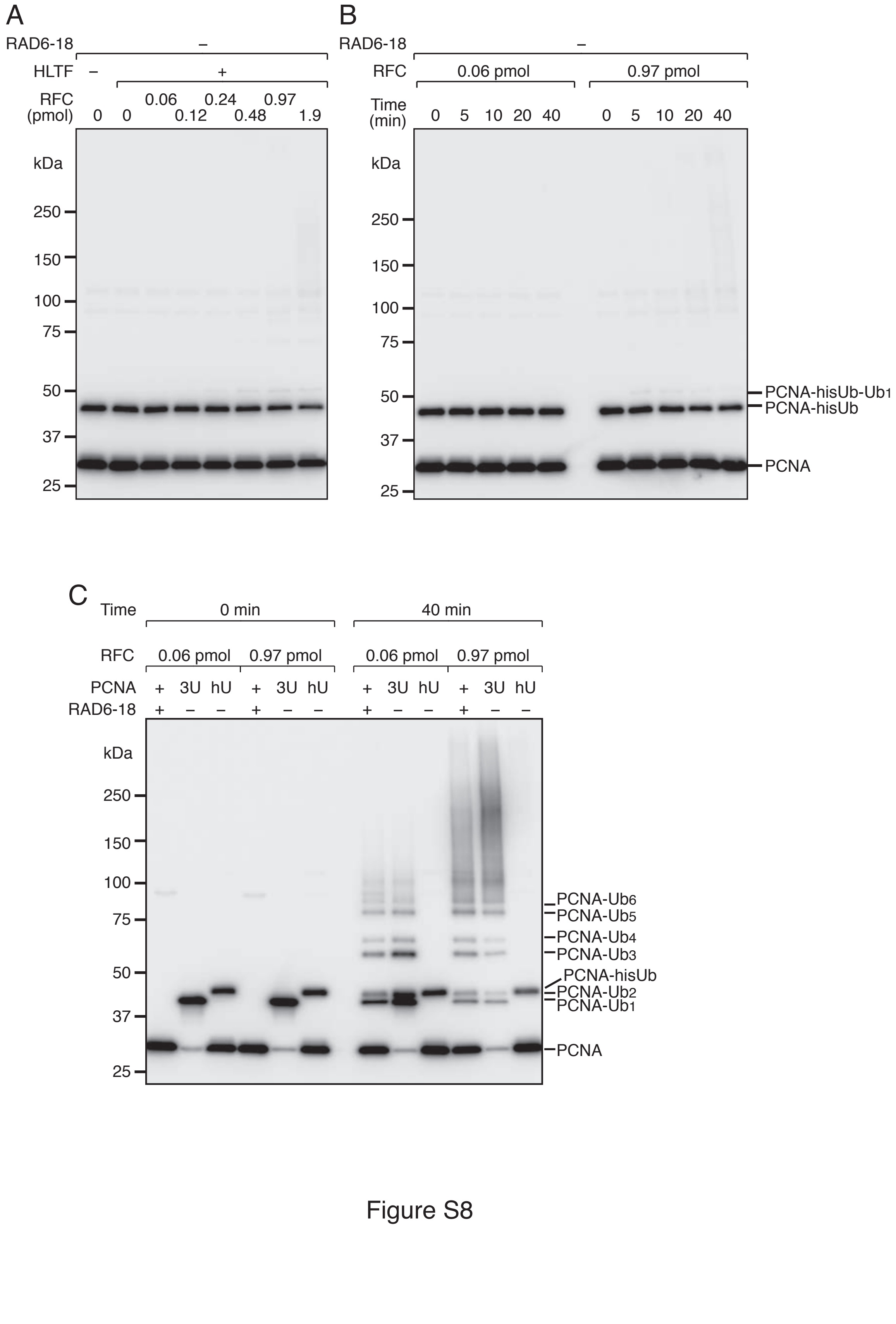
A
RAD6-18
HLTF
RFC (pmol)
0 0 0.06 0.12 0.24 0.48 0.97 1.9
kDa
250
150
100
75
50
37
25
B
Time (min)
0.06 pmol
0.97 pmol
0 5 10 20 40
PCNA-hisUb-Ub1
PCNA-hisUb
PCNA
C
Time
0 min
40 min
RFC
PCNA
3U
hU
PCNA-Ub6
PCNA-Ub5
PCNA-Ub4
PCNA-Ub3
PCNA-Ub2
PCNA-Ub1

Figure S8
